# 10 years of mindlines: a systematic review and commentary

**DOI:** 10.1186/s13012-015-0229-x

**Published:** 2015-04-09

**Authors:** Sietse Wieringa, Trisha Greenhalgh

**Affiliations:** Centre for Primary Care and Public Health, Barts and The London School of Medicine and Dentistry, Queen Mary University of London, 58 Turner Street, London, E1 2AB UK; Department of Primary Care Health Sciences, New Radcliffe House (2nd floor), Walton Street, Oxford, OX2 6GG UK

**Keywords:** Mindlines, Tacit knowledge, Knowledge in practice, Knowledge translation, Knowledge creation, guidelines, EBM, Evidence-based medicine, Meta-narrative review, Systematic review

## Abstract

**Background:**

In 2004, Gabbay and le May showed that clinicians generally base their decisions on mindlines—internalised and collectively reinforced tacit guidelines—rather than consulting written clinical guidelines. We considered how the concept of mindlines has been taken forward since.

**Methods:**

We searched databases from 2004 to 2014 for the term ‘mindline(s)’ and tracked all sources citing Gabbay and le May’s 2004 article. We read and re-read papers to gain familiarity and developed an interpretive analysis and taxonomy by drawing on the principles of meta-narrative systematic review.

**Results:**

In our synthesis of 340 papers, distinguished between authors who used mindlines purely in name (‘nominal’ view) sometimes dismissing them as a harmful phenomenon, and authors who appeared to have understood the term’s philosophical foundations. The latter took an ‘in-practice’ view (studying how mindlines emerge and spread in real-world settings), a ‘theoretical and philosophical’ view (extending theory) or a ‘solution focused’ view (exploring how to promote and support mindline development). We found that it is not just clinicians who develop mindlines: so do patients, in face-to-face and (potentially) online communities.

Theoretical publications on mindlines have continued to challenge the rationalist assumptions of evidence-based medicine (EBM). Conventional EBM assumes a single, knowable reality and seeks to strip away context to generate universal predictive rules. In contrast, mindlines are predicated on a more fluid, embodied and intersubjective view of knowledge; they accommodate context and acknowledge multiple realities. When considering how knowledge spreads, the concept of mindlines requires us to go beyond the constraining notions of ‘dissemination’ and ‘translation’ to study tacit knowledge and the interactive human processes by which such knowledge is created, enacted and shared. Solution-focused publications described mindline-promoting initiatives such as relationship-building, collaborative learning and thought leadership.

**Conclusions:**

The concept of mindlines challenges the naïve rationalist view of knowledge implicit in some EBM publications, but the term appears to have been misunderstood (and prematurely dismissed) by some authors. By further studying mindlines empirically and theoretically, there is potential to expand EBM’s conceptual toolkit to produce richer forms of ‘evidence-based’ knowledge. We outline a suggested research agenda for achieving this goal.

**Electronic supplementary material:**

The online version of this article (doi:10.1186/s13012-015-0229-x) contains supplementary material, which is available to authorized users.

## Background

Ten years ago, Gabbay and le May published a prominent article in the British Medical Journal in which they challenged the ‘over-rationalist model implicit in evidence based health care’ [1,2]. In an ethnographic study in UK general practice, they showed that clinicians only rarely accessed research findings, clinical guidelines and other types of formal knowledge directly. Rather, they preferred to rely on what they called ‘mindlines’, defined as ‘collectively reinforced, internalised tacit guidelines, which were informed by brief reading, but mainly by their interactions with each other and with opinion leaders, patients, and pharmaceutical representatives and by other sources of largely tacit knowledge that built on their early training and their own and their colleagues’ experience’ [1].

Much subsequent research in mainstream health services research pursued and confirmed the negative finding of this study—that doctors rarely consult written guidelines when making clinical decisions. Studies sought to identify ‘barriers’ to guideline implementation on the assumption that more assiduous following of guidelines by individuals would lead to more evidence-based care—see for example [3-5]. A somewhat smaller literature (reviewed below) aligned with Gabbay and le May’s positive finding—that doctors follow mindlines—and sought to characterise, explore and occasionally critique the concept of collectively embodied tacit knowledge and how it links to the goal of evidence-based practice.

The early evidence-based medicine movement (more commonly known as EBM, but also referred to as evidence-based healthcare or practice) explicitly set out to ‘de-emphasise intuition, unsystematic clinical experience, and pathophysiologic rationale as sufficient ground for clinical decision making’, whilst stressing the instrumental role of evidence from research (especially the ‘gold standard’ design, the randomised controlled trial) in clinical decision-making [6]. Although Sackett et al. later softened this stance by writing that ‘evidence based medicine is not restricted to randomised trials and meta-analyses’ and should be seen as ‘the best external evidence with which to answer our clinical questions’ [7], at a philosophical level, EBM seems to rest on a Cartesian view of knowledge as ‘facts’ that are stored in the heads of individual practitioners and/or in formal knowledge repositories, separate from the physical body, independently verifiable and distinct from values. Notwithstanding recent calls by the ‘real EBM’ campaign for a broadening of the parameters of EBM [8], such a conceptualisation would see mindlines (at best) as a lesser form of knowledge and (at worst) as not really existing.

In this paper, we want to discuss *whether* and *how* mindlines have influenced, or should influence, the EBM movement. To explore the impact of mindlines on EBM, we sought to document how the concept of mindlines was picked up and applied by researchers and practitioners following Gabbay and le May’s seminal paper. We also sought to further advance how mindlines have challenged EBM by drawing together findings from these papers as well as revisiting Gabbay and le May’s detailed explanation of mindlines in their 2011 book and bringing in relevant literature from a wider range of disciplinary traditions, including philosophy of science. In this review, we argue that to study mindlines to their full potential, we need to break out of the constraining notions of ‘dissemination’ and ‘translation’ (both of which imply a Cartesian view of knowledge) and focus more on the embodied nature of tacit knowledge and the interactive processes of knowledge creation.

## Methods

We started by searching for any article with a reference to the word mindline or mindlines using the search query mindline* in the PubMed, Web of Science and OvidSP reference search engines. In PubMed, we selected all NCBI databases up to 2014 and in Ovid the Embase, 1996 to 2014 Week 50, HMIC Health Management Information Consortium 1979 to November 2014, Journals@Ovid Full Text December 16, 2014 and Books@Ovid December 11, 2014 databases. We then searched and included articles that cited Gabbay and le May’s 2004 paper on Web of Science and screened Google scholar and books for any further relevant references. We merged the results and excluded hits in which the term ‘Mindline’ was an author’s name.

### Data analysis

We undertook an interpretive synthesis of this literature, drawing on the principles of meta-narrative review [9,10]. This approach was selected because it is designed to capture different underlying assumptions about, and approaches to, a concept. In an initial familiarisation phase, we independently read the articles repeatedly and highlighted sections where the term ‘mindline(s)’ was used. We then collaboratively sorted the articles into paradigmatic groups (i.e. by the philosophical assumptions, theoretical models and methodological approaches shared by different groups of authors). In a subsequent synthesis phase, we compared and contrasted these different paradigmatic perspectives using narrative methods.

In explaining our findings, we adapted a taxonomy originally developed by Orlikowski and Iacono [11] to classify the different ways in which the term ‘IT [information technology]’ was used in papers describing ‘IT research’. The reason for this was that the notion of the ‘nominal’ view from this taxonomy applied very well to many papers in our sample (i.e. papers in which mindlines were mentioned by name but never defined or explored). To this, we added an ‘in practice’ view (i.e. observational studies of how mindlines are enacted in real-world clinical practice), a ‘theoretical and philosophical’ view (in which mindlines were included in a wider theory of knowledge sharing and/or collaborative practice or articles discussing the ontological and/or epistemological assumptions of the concept of mindlines), and a ‘solution’ view (i.e. proposals for how the development of evidence-based mindlines might be promoted and supported). Importantly, many articles could be incorporated into several of these views and these framings are not intended to be mutually exclusive or fixed beyond debate.

## Results

Searching for mindline(s), we found one book, 139 articles in PubMed Central (PMC), 11 articles in PubMed, 14 results in Web of Science and 69 results in Ovid. When merged, 196 results remained. We found 213 results citing Gabbay and le May’s paper and another 24 publications by screening Google scholar and books. After merging these results, removal of duplicates and excluding 12 articles by or referring to authors named Mindline, 340 references remained. The complete reference list is in Additional file 1.

There were no publications before 2004 in the remaining dataset; Gabbay and le May appear to be the first researchers to use the word ‘mindline’ [1]. They subsequently elaborated on the concept in a book [12]. Although the paradigm-shifting potential of the minxsgrdlines concept was the subject of an early BMJ editorial [2], uptake of the term by the wider academic community was slow. A study from 2006 was unable to detect ‘mindline’ as a term in a large sample of articles from 12 major medical journals [13]. Most papers referred to Gabbay and le May’s work on mindlines in an implicit way (for example, in 195 publications, we were able to confirm that the term ‘mindline(s)’ was not used at all).

Literature on mindlines that appeared after Gabbay and le May’s article could broadly be categorised into four groups representing different (but overlapping) paradigmatic perspectives. First, there was the ‘nominal’ framing, in which authors referred (implicitly or explicitly) to mindlines but did not further explain or expand. We found 133 papers that would primarily fit this view. For instance, mindlines are mentioned in this paper as an unelaborated example of sub-optimal practice when the president of the Australian Patient Safety Foundation is quoted:

*‘There’s a tendency to criticize evidence in order to maintain the status quo, […] medical practice is currently dictated by traditional approaches and “collective mindlines”.’* [14]*.*

In seven papers, comments appeared to come from the guideline development community, and most of them best fitted the nominal view. For example, a paper whose first author’s affiliation is the Italian Cochrane centre, mindlines are referred to as ‘anachronistic’ [15]. Another paper from the Canadian Thoracic Society (erroneously in our view) conflates mindlines with the rapid exchange of ‘easily understood’ information among practitioners:

*‘The literature suggests that health care providers seldom consult guidelines in practice; instead, they rapidly glean pieces of information from documents or colleagues with whom they construct “mindlines” that inform clinical decisions. To emulate this practical knowledge exchange medium, guidelines should include information that can be easily understood and transmitted’* [16].

We believe this recommendation reflects a naïve conceptualisation of the ontology of knowledge (seen as facts to be transmitted, simple ones being more transmissible than complicated ones). As we argue below, to understand mindlines requires that we go beyond such a conceptualisation.

The second framing of mindlines, we gleaned from the literature was the ‘in practice’ view, in which the term was used to explain the empirical finding that clinicians are rarely observed to follow written guidelines (but appear to follow mindlines instead). We found 76 papers that primarily referred to mindlines from this point of view. In these papers, the term ‘mindlines’ often seemed to mean ‘consulting colleagues’. For example, one study showed that, like GPs, general surgeons most often turned to colleagues before using other sources of knowledge such as the internet, educational meetings or the library [17].

In a few studies, the ‘in practice’ view of mindlines closely reflected Gabbay and le May’s original theorisation. In a study on the role of tacit knowledge in how public healthcare groups planned initiatives, for example, the authors found that *‘study participants used collectively reinforced tacit guidelines based on experiences and interactions in fluid communities of practice rather than drawing on research findings or explicit practice guidelines’* [18]. In a study on the influences on prescribing in general practice, Grant et al. found that practitioners used ‘prescribing mindlines’. *‘These were personal formularies developed from and informed by their experience of medication (including patient’s experiences), specialist advice, discussions with their practice pharmacist and GP colleagues, and the practice’s macro prescribing policy (if present). GPs rarely looked up information about medicines and relied on these prescribing mindlines. […] GPs relied on personal experience and social networks to update their mindlines’* [19].

In a cross-national study on what physicians gain and lose with clinical experience with diabetes, Elstad et al. write *‘In tune with Gabbay & le May’s “mindlines” […], we found that physicians gained their experience in part through their professional interactions’*. They then quote an experienced German clinician who comments on comparing intuitive concerns: *‘Happily we are two physicians working here… I say, “It’s funny, something is wrong with him”. And my colleague says, “You know, you are right. Something is wrong. He simulated, or lied or something is rotten”. We nearly always agree. You can’t have that in an office where you work alone, but it’s really good. We swap ideas on the patients’* [20]*.*

Gabbay’s and le May’s ethnographic methodology is replicated in one of the few studies in the literature to tease out *what the key mindlines were* in a particular topic area [21]. In this qualitative study, the authors invoke three ‘mindlines’ of clinicians which appear to explain why malaria is overdiagnosed in Tanzania. In this example, however, one could claim that mindlines are merely depicted as unspoken rules of thumb or maxims that generally override more specific and detailed formal recommendations:

*‘Rather than following national guidelines for the diagnosis of febrile illness, clinician behaviour appeared to follow “mindlines”: shared rationales constructed from these different spheres of influence. Three mindlines were identified in this setting: malaria is easier to diagnose than alternative diseases; malaria is a more acceptable diagnosis; and missing malaria is indefensible. These mindlines were apparent during the training stages as well as throughout clinical careers.’* [21]

Chandler et al.’s study is cited in 33 papers in our sample, but most of these do not actually refer to the concept of mindlines (they cite the paper to support the statement that there is overdiagnosis of malaria).

Third, mindlines were sometimes framed as a theoretical or philosophical concept in publications (with or without an empirical component) whose main purpose appeared to be the development of theory. We classified 57 papers as predominantly this framing.

Many theoretical papers discussed mindlines in the light of Lave and Wenger’s theory of communities of practice. For instance, Li et al. combine the concept of mindlines with communities of practice and Nonaka and Takeuchi’s knowledge creation cycle (which considers how formal codified knowledge is made tacit and disseminated among organisational members through observation and discussion, thereby becoming meaningful and applicable in practice) [22]. They state:

*‘Explicit knowledge is codified information such as peer-reviewed articles, rules, and guidelines, which can be readily shared among people. However, to apply this knowledge in practice, practitioners must make sense of the concrete information in the context in which it is used. This process of establishing meaning can be facilitated by discussions with colleagues and mentors or by observing how others apply the knowledge and then try it themselves.’* [22]

Similarly, Crites et al. talk about the knowledge creation cycle and link this to the literature on learning organisations as described by Argyris and Schon [23]; they take from mindlines that external knowledge needs to be *‘validated through informal team discourse and modified for practical application’* by working teams [24]. Ranmuthugala includes Gabbay and le May’s ethnography in an elaborate systematic literature review on why and how communities of practice are established in healthcare [25]. Soubhi et al. offer a theorisation of the communities of practice literature, enhanced by the notion of mindlines, as it might be applied to multi-disciplinary care for complex multi-morbidity [26].

Several studies see mindlines as empirical support for the tenets of social network theory primarily by taking the notion from Gabbay and le May’s work that clinicians rely on their peers to acquire knowledge. They set out to explore what personal relationships between clinicians exist—for example, to study the propensity towards EBM of physicians in relation to their ‘coreness’ (that is, closeness to the centre) in their social network [27], to explore how the structure of patient-sharing relationships among physicians [28] is related to care patterns of high or low costs within hospitals [29] and what factors affect the influence of certain physicians in a network on the thinking and practice of other physicians [30]. An alternative argument is that the concept of social networks neither confirms nor refutes the kinds of knowledge exchange that are implicit in the concept of mindlines.

Other studies link mindlines to social influence. Lomas, for example, provides a useful overview of the literature on the cultural gap between research and policymaking—and the essentially *social* (not technical) nature of successful efforts to bridge this gap [31]. He uses the concept of mindlines to underline the social nature of collective influence in both the research and policymaking communities. Nine publications in our sample explicitly criticised the political, economic and ethical dimensions of how research knowledge is generated (for example the tainting of research funding through industry conflicts of interest). However, they usually did not link these critiques to mindlines directly.

In an article on clinical decision-making in Ghana, the authors used a novel theoretical framework of guidelines (explicit), clinicians’ mindlines (tacit) and ‘clientlines’. The last are a patient version of mindlines, consisting of *‘client influences related to the preferences and pressures of the client and the wider family and community, including social, religious and cultural values and beliefs’* [32].

Papers that addressed the ontology (what is it) and epistemiology (how might we study it) of knowledge considered topics such as the difference between explicit and tacit knowledge, the nature of clinical reasoning and the validity and transferability of medical knowledge. For example, several authors referred to mindlines as a form of system 1 (intuitive) as opposed to system 2 (analytical) thinking in articles on clinical information processing and cognitive errors [33-35]. Bate et al. explain:

*‘Dual process theory states that humans process information in two ways, termed System 1 and System 2. System 1 processing is an “intuitive, automatic, fast, frugal and effortless” process, involving the construction of mental maps and patterns, shortcuts and rules of thumb (heuristics), and “mindlines” (collectively reinforced, internalized tacit guidelines). These are developed through experience and repetition, usually based on undergraduate teaching, brief written summaries, seeing what other people do, talking to local colleagues and personal experience. System 2 processing involves a careful, rational analysis and evaluation of the available information. This is effortful and time consuming. Data from a variety of environments demonstrates that human beings prefer to use System 1 processing whenever possible.’* [35]

In a number of papers, however, this deeper understanding of mindlines was missing, and authors simply equated mindlines to heuristics.

Walach et al. propose a ‘circular’ model for linking evidence and clinical decision-making [36,37]. Like Henry et al. [38], they reject the hierarchy of evidence (randomised trials at the top, ‘anecdotes’ at the bottom) as valid only in relation to simple decisions about the efficacy of drug therapies. Most clinical decisions, they argue, are complex; they involve ethical and human decisions as well as scientific ones and, hence, require the integration of multiple considerations and forms of evidence. The tacit knowledge of mindlines may be more appropriate for the complex and organic nature of real-world medicine than the ‘if…then’ structure of guidelines and decision support tools.

In a systematic review of knowledge exchange mechanisms, Contandriopoulos and colleagues consider knowledge in two essential forms: individual, that is, held in people’s heads and translated (or not) into action by human will and agency and collective, that is, socially shared and organizationally embedded—a form akin to Gabbay and le May’s mindlines [39].

A final framing of the ‘mindlines’ concept, sometimes offered as a conclusion in an empirical or theoretical paper, was solution-focused: some authors considered the question of how to actively promote and support the development of valid embodied/collective knowledge or ‘evidence-based mindlines’. In 28 sources, this seemed the main purpose of the article. For example, the challenge was expressed (somewhat obliquely) in a letter by Glasziou, in which he recognises the concept of mindlines in clinical practice, but is worried that they could *‘supply counterfeit evidence. […] a puzzle remains: how do we get valid memes into the mindlines while not driving out the wisdom of experience?’* [40].

Two studies describe efforts to set up (and influence the behaviour of) communities of practice among researchers [41] and doctors [42] by employing a facilitator and co-ordinator respectively, though each of these studies mentions mindlines only in passing. In a more theoretically informed paper, Soubhi et al.’s model of communities of practice in multi-morbidity care is also solution-focused, emphasising relationship-building and collaborative learning as the basis for developing mindlines. They hope that qualitative and quantitative research could *‘examine how primary care physicians develop mindlines and how they test them to eliminate harmful ones and standardize others into routine practice’*.

Others call for knowledge brokers [31], transformational leaders [43], thought leaders [44] and individuals more generally [41] to alter, expand and embed new knowledge through social influence within (and indeed extending beyond) existing communities of practice. These proposals echo and flesh out Gabbay and le May’s original exhortation to make sure that the knowledge circulating within communities is based on sound research [1]. In their systematic review, Contandriopoulos et al. suggest that knowledge may become collectivised through a variety of mechanisms, including efforts to make it relevant (timely, salient, actionable), legitimate (credible, authoritative, reasonable), and accessible (available, understandable, assimilable) and to take account of the assumptions and priorities of a particular audience [34].

Reeve et al. draw parallels between mindlines and the (subtle and often overlooked) skills of expert generalism in GP practice [45]. These authors offer a four-phase approach to developing generalist expertise: sense-making (popularising the concept of generalist expertise and raising awareness of it across a community of practitioners), engagement (influencing practitioners to prioritise this issue), action (e.g. delivering education, promoting scholarship as part of professional practice) and monitoring (measuring the impact of this approach).

In an observational study to assess how ‘evidence-based’ GP consultations were, Zwolsman et al. observed that GPs were often unable to account for the source of their knowledge (personal experience or research evidence) and that rapid, intuitive decisions, suggesting a predominantly tacit form of knowledge, were the norm [46]. The authors felt that making all steps in the decision-making chain more explicit (for example, justifying the chain of reasoning to the patient) would surface gaps in knowledge, inform further learning and—in the long run—make practice more evidence based.

In contrast, Levine et al. suggest the opposite. They propose making guidelines more *implicit* by transforming them in to aphorisms which they define as *‘succinct sayings that offer advice’*. These should function as *‘interface between intuitive approaches to make rapid decisions, and the implementation of specialty-specific clinical guidelines’* [47]*.*

## Discussion

This systematic narrative review has revealed a relatively sparse literature on the important concept of mindlines, first introduced in 2004 by Gabbay and le May. Whilst we included 340 publications in our final dataset, some authors appeared to have (with the best of intentions) used this term incorrectly and naively. But others had recognised and explored mindlines’ fundamental philosophical challenge to EBM. The relatively limited contributions from the guideline development community suggest that the concept has been largely ignored by the guideline industry—perhaps because, as American physicist and philosopher of science Thomas Kuhn wrote, *‘the proponents of competing paradigms practice their trades in different worlds’* [48].

‘Knowledge translation’, one-way transfer of knowledge from producer (research) to user (clinician) [49,50], remains a (contested) policy challenge*.* Multiple research traditions have contributed to a vast body of literature on how evidence from research is disseminated [51,52]. Underlying this literature is a fundamental hope for the possibility of optimising the ‘intermediation’ of knowledge—that is, the managed processes by which practitioners can be supported to interact with knowledge [53]. But an alternative metaphor that goes beyond the concept of ‘spreading’ good ideas is needed [54].

In their book from 2011, Gabbay and le May explain in great detail the origins of mindlines, their implications and related theories [12]. Mindlines fit a view that knowledge is not so much a set of external facts that are waiting to be ‘translated’ or ‘disseminated’ but a more fluid and multi-directional phenomenon in which knowledge is ‘re-created’ in different contexts by different people again and again as previously postulated by Mol and Nonaka [55,56]. From this perspective, improving knowledge intermediation is more like maximising the opportunity to *create* knowledge. How this might be achieved differs from the EBM paradigm in a number of ways uncovered by Gabbay and le May throughout their book. We explore these philosophical questions further below.

### Reality—single or multiple?

Firstly, mindlines question our assumptions about reality. Without doing full justice to the vast literature and on-going debate on the philosophy of knowledge, we acknowledge in particular the philosophical difference between naïve realism (in which there is one reality, which exists independently of human thought and can ultimately be known by everyone) and more pluralist philosophies (such as subjective idealism and critical realism) which assume multiple realities, none of which are fully shared [57].

The possibility of multiple realities explains why different national guidelines for even simple conditions like urinary tract infections draw different conclusions from the latest research [58]. Naïve rationalists (some protagonists of EBM, for example) think that we will overcome these differences and ultimately have a clear set of ‘facts’, recommendations, international clinical guidelines and policies that everyone except the misguided and ignorant will all agree upon. In contrast, as Gabbay and le May point out [12], the concept of mindlines presents us with the idea of a shared (but by no means homogeneous) reality consisting of multiple very individual and temporary realities of people: clinicians, researchers, guideline makers and patients. Mindlines offer a view that the number of guidelines on any topic will never cease to expand because we expect new individual realities and scientific paradigms to emerge continuously.

### The nature of knowledge

Absent in the conventional EBM paradigm, but very present in the concept of mindlines, is the acknowledgement that, to use Polanyi’s phrase, *‘we know more than we can tell’*—that is, not all knowledge is conscious or explicit [59]. Gabbay and le May discuss how mindlines include ‘knowledge-in-practice-in-context’: practical knowledge formed not only by the setting but also by the need for that knowledge [12]. Conventional EBM views knowledge narrowly as factual data, only a tiny fraction of which are relevant to the decision at hand (and that it will be largely self-evident which ‘facts’ are needed). The mindlines concept envisions a ‘negotiating space’ [12] where clinical decision-making by clinicians and patients involves a process of reduction and prioritisation from a vast realm of potentially relevant knowledge of different kinds.

In this sense, we would argue that mindlines stress both the act of and the need for reducing possible options for action. EBM does not reject the idea of reducing knowledge—for example, the guideline development process encourages the dismissal of evidence and knowledge that is considered of low quality [60,61]. But in mindlines, the process of reduction applies not only to explicit knowledge. All kinds of knowledge are competing for attention [12,39]. In clinical consultations, not everything is or can be taken into account; there is limited time [62], our brains do not process everything [35], we are forgetful and the ‘whole’ story is not told to us. Reduction is an essential and ever-present process to create knowledge and experience reality. As Heisenberg wrote *‘what we observe is not nature in itself but nature exposed to our method of questioning’* [63]. Arguably, what we observe as clinicians is not reality itself but the reality exposed to our method of reducing or filtering the various potentially relevant streams of knowledge of which we are consciously or unconsciously aware and from those, constructing a picture of current reality.

As we explore a clinical case by questioning, examining and testing, some things get more ‘certain’, but the overall picture will not necessarily become clearer. For example, in the case of abdominal pain, a stool sample makes us more certain about the organisms involved. However, it says little about the social context in which a particular type of pain occurs and recurs. If we ask a patient about her daily life, we may build a fuller, clearer and more holistic understanding of the abdominal pain—and the stool sample may become less of a priority. Indeed, an incidental growth of a pathogen in the stool sample may confuse and distract us if the ‘real’ cause of the abdominal pain is migraine, marital stress or the side effects of medication. Reducing in the spirit of mindlines is a creative process, not a reductionist one, like carving a particular ‘abdominal pain’ out of a piece of buzzing, blooming reality [64] with an infinite number of dimensions.

### How the ‘truth’ is arrived at

Gabbay and le May offer many detailed examples of how clinicians co-construct knowledge and discuss several theories that help to understand these processes such as the knowledge creation cycle developed by Nonaka and Takeuchi explaining the growth of tacit knowledge in organisations [12]. But unlike the papers we found that simply see mindlines as heuristics, Gabbay and le May explain that logical thinking is embedded in mindlines. What we derive from this is that the set of tools used to reduce or construct (depending on our perception of realities) the knowledge base for a clinical decision differs dramatically between conventional EBM and mindlines. In the former, the dominant tool for identifying knowledge is rational, conscious questioning, and the main requirement of that knowledge is validity—that is, if it fulfils the criteria of correspondence (to the real world—for example, through a robust sampling procedure), coherence (with what we define as a logical system of high-quality knowledge—for example, derived from a well-conducted randomised trial and meeting the standards of statistical prediction) and consensus (experts agree—for example, through peer review). The literature on mindlines, as reflected for instance in the circular evidence model suggested by Walach et al. [36], fundamentally challenges these philosophical criteria by which a finding will become classified as ‘true’ as they are inadequate to make predictions in the real world of clinical practice.

In particular, EBM intentionally focuses on so-called ‘frequentist’ reasoning, in its quest to discard mechanism-based reasoning and reliance on (potentially unreliable) clinical expertise [65,66]. This type of reasoning is an evasion of the fundamental inability to predict the future—or more precisely the problem of induction [67]. If we look at an association between A (such as a taking a tablet) and B (such as a clinical outcome) in the rich context of everyday practice, we may discover a web of interacting influences linking A with B. Evidence-based reasoning is essentially a process of stripping the causal net (compare Pearl [68]) from the association between A and B (‘bias’), in order to find a single general yet virtual rule—and then applying this rule to predict the future in another situation where A and B, but also the bias, are present. This might be termed reality-to-rule-to-reality reasoning. In contrast, mindlines allow other evasions of the induction problem (such as Bayesian learning from a one-off experience [67]) in a chain of reasoning that might be termed reality-to-pattern-to-reality, which allows practitioners to keep the network of causality intact from one case to the next.

As articulated in the idea of systems 1 and 2 knowledge [35], mindlines draw more on tacit knowledge, the knowledge we subconsciously use when focusing on the things we want to do [69]. Subconsciously knowing how to interpret gestures, smell, interaction, environment and time during a consultation reduces prevailing uncertainties and helps us to further shape our holistic understanding and make predictions of what is likely to happen in *this* case.

More importantly, mindlines encapsulate a more sophisticated and comprehensive concept of truth than traditional EBM. As Gabbay and le May eloquently explain [12], mindlines take a constructivist approach to knowledge, assuming that it is created in social processes, through discourse, influenced by cultural and historic forces. This chimes with the work of the Russian philosopher Mikhail Bakhtin, who proposed that a unified truth involves sharing personal knowledge with others who provide a separate perspective. *‘The idea lives not in one person’s isolated individual consciousness—if it remains there only, it degenerates and dies. The idea begins to live, that is, to take shape, to develop, to find and renew its verbal expression, to give birth to new ideas, only when it enters into genuine dialogic relationships with other ideas, with the ideas of others’* [70]. Similarly, the Austrian philosopher Ludwig Wittgenstein argued by the same reasoning that all knowledge is collective [71].

Aligning with this focus on the intersubjective nature of knowledge creation [72,73], we contend that contrary to the fears expressed by Glasziou in the quote above, mindlines are not void of validation processes in spite of being mainly tacit. They convey strong and rich elements of shared sense-making (and hence consensus-making), both conscious and unconscious; they address correspondence with reality as it pushes back in the local context; and they address coherence using other types of evasions of the induction problem. In sum, mindlines can be accurate and useful in a local setting and provide useful predictions, despite not being construed according the set of reduction tools and beliefs underpinning the EBM paradigm.

### Economics, politics and ethics

The political, economic and ethical dimensions of the processes of knowledge creation in the papers in our sample were almost never directly linked to mindlines. This is surprising, given that mindlines and traditional EBM differ considerably in this perspective. Several papers in our sample noted that politicians, research leaders, management consultant firms, lobbying groups, the pharmaceutical industry and many other powerful actors use their influence to define research priorities, what counts as medical evidence, how knowledge is distributed and how resources are allocated [31,39,74-76]. In EBM, population-derived statistical estimates fit the needs of policymakers as they provide truths that are—apparently—‘right’ for groups and those who interact with those groups, such as governments and the pharmaceutical industry. What is viewed as good care for a defined group as a whole is in some way regarded as good for individuals. Mindlines however lack this overarching ‘built-in’ criterion of what is right or wrong patient care. With other authors, Gabbay and le May worry that mindlines can spread ‘collective folly’ [12].

EBM strongly adheres to the ‘deficit model’ [77], which entails that clinicians and patients are regarded as deficient in certain knowledge: evidence-based knowledge. This is considered a moral problem of *‘leaving people incapable of understanding the world around them’* [78]. Mindlines, on the other hand, correspond more with the idea that anyone, including patients, create valid knowledge too and can be ‘experts’ in consultations [79]. With the current evolution towards person-based medicine and practices [80], the deficit model may be set to give way to a more pluralist and constructivist one. But at the same time, this may uncomfortably question our basic assumptions about who decides what is good or bad care.

#### Knowledge management, knowledge intermediation

Although Gabbay and le May aptly remark *‘how ironically inconsistent it would be if [they would] try to dictate how [their] work should be put in practice’* [12], many of the implications they discuss have been explored in the literature we found. We discovered articles that explored which sources of knowledge clinicians actually use, projects that aimed to bring research and practice closer together and the development of organisational structures such as communities of practice and virtual social networks to support the use of ‘knowledge-in-practice-in-context’. But what strikes us when considering these implications for practice is the question of whether controlling knowledge creation is actually feasible. EBM assumes that knowledge can be managed, and that, through intermediation, the knowledge deficit of both practitioners and patients can be rectified.

In contrast, mindlines remind us by their emphasis on tacit knowledge that knowledge creation is in large part unmanageable. A one-off event experienced by an individual is all too real for them, ‘anecdotal’ though it may be to others. Furthermore, we cannot control all interactions nor can we control all aspects of resources and contexts. Knowledge development is an organic rather than rational process, which can only be controlled to a limited extent. Currie et al. note that the implication of mindlines is that *‘any attempt at managing professionalised and tacit knowledge in health care through the mobilisation of explicit and codified knowledge faces significant challenge’* [81]*.* As Contandriopoulos et al. conclude in their review on the dissemination of knowledge: *‘…the quest for context-independent evidence on the efficacy of knowledge exchange strategies is probably doomed’* [39]. This picture of knowledge as fluid, multiple, uncontainable and defying rationality is a long way from the hopes and dreams of the EBM movement [6] or even science itself. Thomas Kuhn touches the core of the matter writing: *‘We are all deeply accustomed to seeing science as the one enterprise that draws constantly nearer to some goal set by nature in advance. But need there be any such goal?’* [48].

However, it would be wrong to conclude that because mindlines are constructed, tacit, emergent and shared, they are directionless. On the contrary, there is evidence that the knowledge of mindlines is ‘self-organising’, tending to achieve stability over time. Broekaert noticed *‘real human commitment consists of an open, methodical, meaningful search for the best solution for a certain problem’* [82]*.* Consciously and subconsciously, we collectively create and continuously refine more or less enduring frameworks to look at the world, based on our previous experiences, opinions of colleagues and experts, practical knowledge, guidelines and articles, produced in discourse, agreement and consensus with others, limited by psychological abilities, contexts and the physical world. The more closely the statement, ‘This patient probably has a viral illness, tomorrow she will be OK, even if she doesn't take antibiotics today’ persists in such a framework, the more real, useful and valid it becomes.

Persistence is not without risk. In evidence-based guideline development and research, we are used to synthesising a single version of reality to settle differences [83] and provide consistency of care. But ‘consistency’ of care can harm as well as help individual patients since such an approach may limited our list of differential diagnoses to common or obvious options, removing the possibility of managing the unusual case differently [84]. Similarly, we have to prevent our mindlines from becoming too rigid. Unanimity, or the absence of logical contradiction, prevents the development of new, competing theories and innovations, which needed from time to time to replace the current paradigms [48].

This raises the question of why persistence of knowledge in the form of mindlines is valuable if knowledge is inherently ephemeral and too much persistence risks making our decisions too rigid. Ernst Mach argued: *‘If our dreams were more regular, more connected, more stable, they would also have more practical importance for us’* [85]. Haridimos Tsoukas contends that *‘[U]nderstanding presupposes an Archimedes’ point, a perspective (undoubtedly an irremediably open-ended and evolving perspective, but a perspective nonetheless) from which the world may be viewed, accounted for, and interpreted. Ironically, abundantly available information leads to formlessness and, thus, to a diminished capacity for understanding.’* [86]. Persistence keeps a perspective open for exploration, prediction and guidance of human behaviour. We need persistence to help us to see and find new events, insights and practices so we limit discontinuity or instability that nobody agrees with. Most of infinite reality is not created, distributed, translated or mediated. We miss things because *we have to* in order to experience anything at all.

Hasok Chang [87] argues that scientific realism should commit to pursue many theories to find where reality ‘resists’, whilst investing to preserve theories that did not seem to work that well. In the future, those might turn out to give helpful alternative insights. Applied to mindlines, this may translate to a call to create a broad menu of mindlines to find where collective reality ‘resists’ using many methods of truth finding. If we want to intermediate the process of knowledge creation (to the limited extent that this is possible), further research needs to look into how to speed up the cycle of building and turning over many more persistent mindlines, whilst keeping alternative, less persistent ones afloat efficiently.

In sum, mindlines offers a philosophically and theoretically sophisticated perspective on knowledge and clinical method. Yet in 10 years since the concept was introduced, the study of mindlines has remained a minority sport within critical social science whilst research within the EBM movement on the generation, circulation and use of evidence has remained predominantly (though by no means exclusively) wedded to a naïve rationalist view of knowledge.

The strength of this review is its tight focus on the word ‘mindlines’ and Gabbay and le May’s original 2004 paper in the literature to address the question of how the word and their work have been used, and the meta-narrative approach which allowed us to consider different philosophical assumptions behind different uses of the word. The limitation is that the concept of mindlines may have been discussed more extensively in grey literature, institutional reports and other forums, which we would not have detected using our search.

We hope this review will encourage practitioners and policymakers, along with academics, to embrace fully the implications of the mindlines paradigm. In our sample, Malterud noticed that the EBM movement does not limit the best evidence to randomised controlled trials (RCTs) and meta-analysis, *‘Yet, the foundation for integrating the available sources of knowledge remains unclear. We still do not know whether convincing information leads to optimal decision making’* [88]. Similarly, Jonas argues that *‘we need to broaden and deepen our understanding of what counts as “evidence” and which types of evidence are best used to inform differing aspects of clinical decision making’* [89]. A new research agenda is needed, which should centre first and foremost on the processes and interactions by which mindlines are validated by both clinicians and patients. Research should also seek to break down the walls between EBM and mindlines, for example, by exploring how mindlines emerge and are negotiated in guideline development groups and research communities. Through such interdisciplinary work, it should be possible to identify ways to broaden the methods that such groups could use to create richer and more valid forms of ‘evidence-based’ knowledge.

## Additional file

Additional file 1:
**The complete reference list.** Merged search engine results of citations until the end of 2014 with a reference to the word mindline or mindlines in PubMed, Web of Science and OvidSP; as well as articles that cited Gabbay and le May’s 2004 paper on Web of Science and further relevant hits in Google scholar and Google books.
