# Supplementary material for: 10 years of mindlines: a systematic review and commentary
Source: Implement Sci. 2015 Apr 9;10:45. doi: 10.1186/s13012-015-0229-x (PMC4399748; doi:10.1186/s13012-015-0229-x)
Supplement: Additional file 1: — The complete reference list. Merged search engine results of citations until the end of 2014 with a reference to the word mindline or mindlines in PubMed, Web of Science and OvidSP; as well as articles that cited Gabbay and le May’s 2004 paper on Web of Science and further relevant hits in Google scholar and Google books. [file 13012_2015_229_MOESM1_ESM.doc]

Additional file 1

1. Abreha T, Alemayehu B, Tadesse Y, Gebresillassie S, Tadesse A, Demeke L, Zewde F, Habtamu M, Tadesse M, Yadeta D, Teshome D, Mekasha A, Gobena K, Bogale H, Melaku Z, Reithinger R, Teka H: **Malaria diagnostic capacity in health facilities in Ethiopia.** *Malar J* 2014, **13**:292.

2. Ackerman S, Gonzales R: **The Context of Antibiotic Overuse**. *Ann Intern Med* 2012, **157**:211.

3. Ali N, Whiddett D, Tretiakov A, Hunter I: **The use of information technologies for knowledge sharing by secondary healthcare organisations in New Zealand**. *Int J Med Inform* 2012, **81**:500–506.

4. Allen D: **From boundary concept to boundary object: The practice and politics of care pathway development**. *Soc Sci Med* 2009, **69**:354–361.

5. Allen LK, Hetherington E, Manyama M, Hatfield JM, van Marle G: **Using the social entrepreneurship approach to generate innovative and sustainable malaria diagnosis interventions in Tanzania: a case study.** *Malar J* 2010, **9**:42.

6. Amin A, Roberts J: **Knowing in action: Beyond communities of practice**. *Res Policy* 2008, **37**:353–369.

7. André M, Andén A, Borgquist L, Rudebeck CE: **GPs’ decision-making--perceiving the patient as a person or a disease.** *BMC Fam Pract* 2012, **13**:38.

8. Angus J: **Ethnographic research applied to anesthesia**. *Can J Anaesthesia-Journal Can D Anesth* 2005, **52**:899–903.

9. Ansah EK, Reynolds J, Akanpigbiam S, Whitty CJM, Chandler CIR: **“Even if the test result is negative, they should be able to tell us what is wrong with us”: a qualitative study of patient expectations of rapid diagnostic tests for malaria.** *Malar J* 2013, **12**:258.

10. Arason VA, Sigurdsson JA, Kristinsson KG, Getz L, Gudmundsson S: **Otitis media, tympanostomy tube placement, and use of antibiotics**. *Scand J Prim Health Care* 2005, **23**:184–191.

11. Arnold LK, Alomran H, Anantharaman V, Halpern P, Hauswald M, Malmquist P, Molyneux E, Rajapakse B, Ranney M, Razzak J: **Knowledge translation in international emergency medical care**. *Acad Emerg Med* 2007, **14**:1047–1051.

12. Ayieko P, Ntoburi S, Wagai J, Opondo C, Opiyo N, Migiro S, Wamae A, Mogoa W, Were F, Wasunna A, Fegan G, Irimu G, English M: **A Multifaceted Intervention to Implement Guidelines and Improve Admission Paediatric Care in Kenyan District Hospitals: A Cluster Randomised Trial.** *PLoS Med* 2011, **8**:e1001018.

13. Baiden F, Owusu-Agyei S, Bawah J, Bruce J, Tivura M, Delmini R, Gyaase S, Amenga-Etego S, Chandramohan D, Webster J: **An Evaluation of the Clinical Assessments of Under-Five Febrile Children Presenting to Primary Health Facilities in Rural Ghana.** *PLoS One* 2011, **6**.

14. Baldwin DS, Talat B: **Should benzodiazepines still have a role in treating patients with anxiety disorders?**. *Hum Psychopharmacol* 2012, **27**:237–8.

15. Baltzell K, Elfving K, Shakely D, Ali AS, Msellem M, Gulati S, Mårtensson A: **Febrile illness management in children under five years of age: a qualitative pilot study on primary health care workers’ practices in Zanzibar.** *Malar J* 2013, **12**:37.

16. Barley M, Pope C, Chilvers R, Sipos A, Harrison G: **Guidelines or mindlines? A qualitative study exploring what knowledge informs psychiatrists decisions about antipsychotic prescribing**. *J Ment Heal* 2008, **17**:9–17.

17. Barnett ML, Christakis NA, O’Malley J, Onnela J-P, Keating NL, Landon BE: **Physician patient-sharing networks and the cost and intensity of care in US hospitals.** *Med Care* 2012, **50**:152–60.

18. Barnett ML, Landon BE, O’Malley AJ, Keating NL, Christakis NA: **Mapping Physician Networks with Self-Reported and Administrative Data.** *Health Serv Res* 2011, **46**:1592–1609.

19. Bastiaens GJH, Bousema T, Leslie T: **Scale-up of malaria rapid diagnostic tests and artemisinin-based combination therapy: challenges and perspectives in sub-Saharan Africa.** *PLoS Med* 2014, **11**:e1001590.

20. Bate L, Hutchinson A, Underhill J, Maskrey N: **How clinical decisions are made.** *Br J Clin Pharmacol* 2012, **74**:614–20.

21. Berg A, Patel S, Langeland N, Blomberg B: **Falciparum malaria and HIV-1 in hospitalized adults in Maputo, Mozambique: does HIV-infection obscure the malaria diagnosis?**. *Malar J* 2008, **7**.

22. Bhattacharyya O, Reeves S, Zwarenstein M: **What Is Implementation Research? Rationale, Concepts, and Practices**. *Res Soc Work Pract* 2009, **19**:491–502.

23. Bissett SM, Stone KM, Rapley T, Preshaw PM: **An exploratory qualitative interview study about collaboration between medicine and dentistry in relation to diabetes management.** *BMJ Open* 2013, **3**.

24. Björnsdóttir I, Kristinsson KG, Hansen EH: **Diagnosing infections: a qualitative view on prescription decisions in general practice over time.** *Pharm World Sci* 2010, **32**:805–14.

25. Blair M: **Getting evidence into practice--implementation science for paediatricians.** *Arch Dis Child* 2014, **99**:307–9.

26. Blundell N, Taylor-Phillips S, Spitzer D, Martin S, Forde I, Clarke A: **Elective surgical referral guidelines - background educational material or essential shared decision making tool? A survey of GPs’ in England.** *BMC Fam Pract* 2011, **12**.

27. Boon MH: **How well are we doing in supporting evidence-based health care? The “Information Mastery” perspective**. *Health Info Libr J* 2005, **22**:290–293.

28. Booth J, Tolson D, Hotchkiss R, Schofield I: **Using action research to construct national evidence-based nursing care guidance for gerontological nursing**. *J Clin Nurs* 2007:945–953.

29. Bordoloi P, Islam N: **A Framework Linking Knowledge Management Practices and Healthcare Delivery Performance**. In *8th ICICKM*. Edited by Ribiere V, Worasinchai L.; 2011:655–663.

30. Bracha Y, Brottman G, Carlson A: **Physicians, Guidelines, and Cognitive Tasks**. *Eval Health Prof* 2011, **34**:309–335.

31. Bradbury H: *Practical Prescribing for Medical Students*. *Volume 8*. SAGE Publications; 2013:239320.

32. Brattheim B, Faxvaag A, Tjora A: **Getting the aorta pants in place: A “community of guidance” in the evolving practice of vascular implant surgery**. *Health (Irvine Calif)* 2011, **15**:441–458.

33. Breen AC, Carr E, Langworthy JE, Osmond C, Worswick L: **Back pain outcomes in primary care following a practice improvement intervention:- a prospective cohort study.** *BMC Musculoskelet Disord* 2011, **12**:28.

34. Breen A, Austin H, Campion-Smith C, Carr E, Mann E: **“You feel so hopeless”: A qualitative study of GP management of acute back pain**. *Eur J Pain* 2007, **11**:21–29.

35. Brito Rocha ES, Nagliate P, Bis Furlan CE, Rocha K, Trevizan MA, Costa Mendes IA: **Knowledge management in health: a systematic literature review**. *Rev Lat Am Enfermagem* 2012, **20**:392–400.

36. Broekaert E, Autrique M, Vanderplasschen W, Colpaert K: **“The Human Prerogative”: A Critical Analysis of Evidence-Based and Other Paradigms of Care in Substance Abuse Treatment**. *Psychiatr Q* 2010, **81**:227–238.

37. Bromley E: **A collaborative approach to targeted treatment development for schizophrenia: A qualitative evaluation of the NIMH-MATRICS project**. *Schizophr Bull* 2005, **31**:954–961.

38. Bryant SL, Gray A: **Demonstrating the positive impact of information support on patient care in primary care: A rapid literature review**. *Health Info Libr J* 2006:118–125.

39. Bucknall T, Forbes H: **There Is No “I” in TEAM: Working Cooperatively to Implement Evidence into Practice**. *Worldviews Evidence-Based Nurs* 2009, **6**:187–189.

40. Buetow S: **Making the Improbable Probable: Communication across Models of Medical Practice**. *Heal Care Anal* 2014, **22**:160–173.

41. Burke JP, Gitlin LN: **How Do We Change Practice When We Have the Evidence?**. *Am J Occup Ther* 2012:e85–e88.

42. Burkiewicz JS, Zgarrick DP: **Evidence-based practice by pharmacists: utilization and barriers.** *Ann Pharmacother* 2005, **39**:1214–9.

43. Butalid L, Bensing JM, Verhaak PFM: **Talking about psychosocial problems: An observational study on changes in doctor-patient communication in general practice between 1977 and 2008**. *Patient Educ Couns* 2014, **94**:314–321.

44. Buykx P, Humphreys J, Wakerman J, Perkins D, Lyle D, McGrail M, Kinsman L: **Making evidence count’: A framework to monitor the impact of health services research**. *Aust J Rural Health* 2012, **20**:51–58.

45. Byng R: **Care for common mental health problems: applying evidence beyond RCTs.** *Fam Pract* 2012, **29**:3–7.

46. Cabitza F, Simone C: **Investigating the role of a Web-based tool to promote collective knowledge in medical communities**. *Knowl Manag Res Pract* 2012, **10**:392–404.

47. Calderón C, Rotaeche R, Etxebarria A, Marzo M, Rico R, Barandiaran M: **Gaining insight into the Clinical Practice Guideline development processes: qualitative study in a workshop to implement the GRADE proposal in Spain.** *BMC Health Serv Res* 2006, **6**:138.

48. Cardini F: *Clinical Research in Complementary Therapies. Principles, Problems and Solutions*. *Volume 12*. Elsevier Health Sciences; 2004:59.

49. Carlsen B: **The last frontier? Autonomy, uncertainty and standardisation in general practice**. *Heal Sociol Rev* 2010, **19**:260–272.

50. Carlsen B, Hole AR, Kolstad JR, Norheirn OF: **When you can’t have the cake and eat it too A study of medical doctors' priorities in complex choice situations**. *Soc Sci Med* 2012, **75**:1964–1973.

51. Castelino RL, Bajorek B V, Chen TF: **Are interventions recommended by pharmacists during Home Medicines Review evidence-based?**. *J Eval Clin Pract* 2011, **17**:104–110.

52. Chabot J.-M.: **From guidelines to mindlines**. *Rev du Prat* 2006, **56**:1117–1118.

53. Chandler CIR, Hall-Clifford R, Asaph T, Pascal M, Clarke S, Mbonye AK: **Introducing malaria rapid diagnostic tests at registered drug shops in Uganda: limitations of diagnostic testing in the reality of diagnosis.** *Soc Sci Med* 2011, **72**:937–44.

54. Chandler CIR, Jones C, Boniface G, Juma K, Reyburn H, Whitty CJM: **Guidelines and mindlines: why do clinical staff over-diagnose malaria in Tanzania? A qualitative study.** *Malar J* 2008, **7**:53.

55. Chandler CIR, Kizito J, Taaka L, Nabirye C, Kayendeke M, DiLiberto D, Staedke SG: **Aspirations for quality health care in Uganda: How do we get there?**. *Hum Resour Health* 2013, **11**:13.

56. Chandler CIR, Meta J, Ponzo C, Nasuwa F, Kessy J, Mbakilwa H, Haaland A, Reyburn H: **The development of effective behaviour change interventions to support the use of malaria rapid diagnostic tests by Tanzanian clinicians.** *Implement Sci* 2014, **9**:83.

57. Chandler CIR, Whitty CJM, Ansah EK: **How can malaria rapid diagnostic tests achieve their potential? A qualitative study of a trial at health facilities in Ghana.** *Malar J* 2010, **9**:95.

58. Chapman NH, Lazar SP, Fry M, Lassere MN, Chong BH: **Clinicians adopting evidence based guidelines: a case study with thromboprophylaxis.** *BMC Health Serv Res* 2011, **11**:240.

59. Chisolm DJ, McAlearney AS, Veneris S, Fisher D, Holtzlander M, Mccoy KS: **The role of computerized order sets in pediatric inpatient asthma treatmentThe role of computerized order sets in pediatric inpatient asthma treatment**. *Pediatr Allergy Immunol* 2006, **17**:199–206.

60. Clark F: **Beyond high definition: Attitude and evidence bringing OT in HD-3D**. *Am J Occup Ther* 2012:644–651.

61. Clark F, Park DJ, Burke JP: **Dissemination: bringing translational research to completion.** *Am J Occup Ther* 2013, **67**:185–93.

62. Clarke A, Blundell N, Forde I, Musila N, Spitzer D, Naqvi S, Browne J: **Can guidelines improve referral to elective surgical specialties for adults? A systematic review.** *Qual Saf Health Care* 2010, **19**:187–94.

63. Colling T, McNinch T, Lighthizer DR: **Assessment of Traffic Safety Training for Local Agency Technical Staff and Elected and Appointed Officials**. *Transp Res Rec J Transp Res Board* 2011, **2213**:63–71.

64. Connell LA, McMahon NE, Harris JE, Watkins CL, Eng JJ: **A formative evaluation of the implementation of an upper limb stroke rehabilitation intervention in clinical practice: a qualitative interview study.** *Implement Sci* 2014, **9**.

65. Contandriopoulos D, Lemire M, Denis J-L, Tremblay E: **Knowledge exchange processes in organizations and policy arenas: a narrative systematic review of the literature.** *Milbank Q* 2010, **88**:444–83.

66. Cramer P: **The EBM Argument Hygiene Campaign**. *J Eval Clin Pract* 2013, **19**:447–453.

67. Crites GE, McNamara MC, Akl EA, Richardson WS, Umscheid CA, Nishikawa J: **Evidence in the learning organization.** *Health Res Policy Syst* 2009, **7**:4.

68. Crump JA, Morrissey AB, Nicholson WL, Massung RF, Stoddard RA, Galloway RL, Ooi EE, Maro VP, Saganda W, Kinabo GD, Muiruri C, Bartlett JA: **Etiology of Severe Non-malaria Febrile Illness in Northern Tanzania: A Prospective Cohort Study.** *PLoS Negl Trop Dis* 2013, **7**.

69. Cullum N, Ciliska D, Haynes B, Marks S (Eds): *Evidence-Based Nursing: An Introduction*. John Wiley & Sons; 2013:336.

70. Curley AL, Vitale P a, Md, Mph, Faap: *Population-Based Nursing: Concepts and Competencies for Advanced Practice*. Springer Publishing Company; 2011:296.

71. Currie G, Burgess N, White L, Lockett A, Gladman J, Waring J: **A qualitative study of the knowledge-brokering role of middle-level managers in service innovation: managing the translation gap in patient safety for older persons’ care**. *Heal Serv Deliv Res* 2014, **2**:1–118.

72. Currie G, Humpreys M, Waring J, Rowley E: **Narratives of professional regulation and patient safety: The case of medical devices in anaesthetics**. *Health Risk Soc* 2009, **11**:117–135.

73. Currie G, Waring J, Finn R: **The limits of knowledge management for UK public services modernization: The case of patient safety and service quality**. *Public Adm* 2008, **86**:363–385.

74. Currin L, Waller G, Treasure J, Nodder J, Stone C, Yeomans M, Schmidt U: **The use of guidelines for dissemination of “best practice” in primary care of patients with eating disorders**. *Int J Eat Disord* 2007, **40**:476–479.

75. Dalsgaard T, Kallerup H, Rosendal M: **Outreach visits to improve dementia care in general practice: a qualitative study**. *Int J Qual Heal Care* 2007, **19**:267–273.

76. Davies C: **Grounding governance in dialogue? Discourse, practice and the potential for a new public sector organizational form in Britain**. *Public Adm* 2007, **85**:47–66.

77. de Pedro Gomez J, Miguel Morales-Asencio J, Sess Abad A, Bennasar Veny M, Ruiz Roman MJ, Munoz Ronda F: **Validation of the Spanish Version of the Evidence Based Practice Questionnaire in Nurses**. *Rev Esp Salud Publica* 2009, **83**:577–586.

78. De Simone J: **Beyond “faith-based medicine” and EBM.** *J Eval Clin Pract* 2006, **12**:438–444.

79. De Souza V, MacFarlane A, Murphy AW, Hanahoe B, Barber A, Cormican M: **A qualitative study of factors influencing antimicrobial prescribing by non-consultant hospital doctors**. *J Antimicrob Chemother* 2006, **58**:840–843.

80. Deo S, Topp SM, Westfall AO, Chiko MM, Wamulume CS, Morris M, Reid S: **Impact of organizational factors on adherence to laboratory testing protocols in adult HIV care in Lusaka, Zambia.** *BMC Health Serv Res* 2012, **12**.

81. Derua YA, Ishengoma DR, Rwegoshora RT, Tenu F, Massaga JJ, Mboera LE, Magesa SM: **Users’ and health service providers' perception on quality of laboratory malaria diagnosis in Tanzania.** *Malar J* 2011, **10**:78.

82. Díaz-Chao A, Torrent-Sellens J, Lacasta-Tintorer D, Saigí-Rubió F: **Improving integrated care: modelling the performance of an online community of practice.** *Int J Integr Care* 2014, **14**:e007.

83. Diogene E, Figueras A: **What public policies have been more effective in promoting rational prescription of drugs?**. *J Epidemiol Community Health* 2011, **65**:387–388.

84. Dixon-Woods M, McNicol S, Martin G: **Ten challenges in improving quality in healthcare: lessons from the Health Foundation’s programme evaluations and relevant literature.** *BMJ Qual Saf* 2012, **21**:876–884.

85. Donner-Banzhoff N, Roth T, Sönnichsen AC, Luckmann J, Leonhardt C, Chenot JF, Becker A, Keller S, Griffithse F, Bauma E: **Evaluating the accuracy of a simple heuristic to identify serious causes of low back pain**. *Fam Pract* 2006:682–686.

86. Douglas F, van Teijlingen E, Torrance N, Fearn P, Kerr A, Meloni S: **Promoting physical activity in primary care settings: health visitors’ and practice nurses' views and experiences**. *J Adv Nurs* 2006, **55**:159–168.

87. Dowding D, Spilsbury K, Thompson C, Brownlow R, Pattenden J: **The decision making of heart failure specialist nurses in clinical practice.** *J Clin Nurs* 2009, **18**:1313–24.

88. Driscoll MP: *Handbook of Research for Educational Communications and Technology: A Project of the Association for Educational Communications and Technology*. Routledge; 2001:1296.

89. Dufault M, Duquette CE, Ehmann J, Hehl R, Lavin M, Martin V, Moore MA, Sargent S, Stout P, Willey C: **Translating an Evidence-Based Protocol for Nurse-to-Nurse Shift Handoffs**. *Worldviews Evidence-Based Nurs* 2010, **7**:59–75.

90. Dugdill L, Crone D, Murphy R: *Physical Activity and Health Promotion: Evidence-Based Approaches to Practice*. John Wiley & Sons; 2009:263.

91. Duggal R, Menkes DB: **Evidence-based medicine in practice**. *Int J Clin Pract* 2011, **65**:639–644.

92. Editorial: **“Mindlines” are still preferred to guidelines.** *BMJ Br Med J* 2004, **329**.

93. Elstad EA, Lutfey KE, Marceau LD, Campbell SM, von dem Knesebeck O, McKinlay JB: **What do physicians gain (and lose) with experience? Qualitative results from a cross-national study of diabetes.** *Soc Sci Med* 2010, **70**:1728–1736.

94. Elwyn G, Taubert M, Kowalczuk J: **Sticky knowledge: a possible model for investigating implementation in healthcare contexts.** *Implement Sci* 2007, **2**:44.

95. English M, Nzinga J, Mbindyo P, Ayieko P, Irimu G, Mbaabu L: **Explaining the effects of a multifaceted intervention to improve inpatient care in rural Kenyan hospitals -- interpretation based on retrospective examination of data from participant observation, quantitative and qualitative studies.** *Implement Sci* 2011, **6**:124.

96. European Commission: **Sustainable and responsible business**. Norwegian University of Science and Technology; 2000.

97. Evans DW, Breen AC, Pincus T, Sim J, Underwood M, Vogel S, Foster NE: **The effectiveness of a posted information package on the beliefs and behavior of musculoskeletal practitioners: the UK Chiropractors, Osteopaths, and Musculoskeletal Physiotherapists Low Back Pain ManagemENT (COMPLeMENT) randomized trial.** *Spine (Phila Pa 1976)* 2010, **35**:858–66.

98. Evans DW, Foster NE, Underwood M, Vogel S, Breen AC, Pincus T: **Testing the effectiveness of an innovative information package on practitioner reported behaviour and beliefs: the UK Chiropractors, Osteopaths and Musculoskeletal Physiotherapists Low back pain ManagemENT (COMPLeMENT) trial [ISRCTN77245761].** *BMC Musculoskelet Disord* 2005, **6**:41.

99. Everitt S: **Applying evidence-based veterinary medicine**. *In Pract* 2008, **30**:526–528.

100. Fattore G, Frosini F, Salvatore D, Tozzi V: **Social network analysis in primary care: The impact of interactions on prescribing behaviour**. *Health Policy (New York)* 2009, **92**:141–148.

101. Faucher J-F, Makoutode P, Abiou G, Béhéton T, Houzé P, Ouendo E, Houzé S, Deloron P, Cot M: **Can treatment of malaria be restricted to parasitologically confirmed malaria? A school-based study in Benin in children with and without fever.** *Malar J* 2010, **9**:104.

102. Feifer C, Ornstein SM, Jenkins RG, Wessell A, Corley ST, Nemeth LS, Roylance L, Nietert PJ, Liszka H: **The logic behind a multimethod intervention to improve adherence to clinical practice guidelines in a nationwide network of primary care practices**. *Eval Health Prof* 2006, **29**:65–88.

103. Ferlie E, Crilly T, Jashapara A, Peckham A: **Knowledge mobilisation in healthcare: A critical review of health sector and generic management literature**. *Soc Sci Med* 2012, **74**:1297–1304.

104. Fernandez RS, Davidson P, Griffiths R, Salamonson Y: **Overcoming barriers to guideline implementation: the case of cardiac rehabilitation**. *Qual Saf Health Care* 2010, **19**:e15.

105. Fernando D, de Silva NL, Ackers I, Abeyasinghe R, Wijeyaratne P, Rajapakse S: **Patient satisfaction and uptake of private-sector run malaria diagnosis clinics in a post-conflict district in Sri Lanka.** *BMC Public Health* 2014, **14**.

106. Fharm E, Rolandsson O, Johansson EE: **“Aiming for the stars”-GPs’ dilemmas in the prevention of cardiovascular disease in type 2 diabetes patients: focus group interviews**. *Fam Pract* 2009, **26**:109–114.

107. Finn R, Waring J: **Organizational Barriers to Architectural Knowledge and Teamwork in Operating Theatres**. *Public Money Manag* 2006, **26**:117–124.

108. Ford EW, Menachemi N, Phillips MT: **Predicting the adoption of electronic health records by physicians: When will health care be paperless?**. *J Am Med Informatics Assoc* 2006, **13**:106–112.

109. Ford EW, Menachemi N, Peterson LT, Huerta TR: **Resistance Is Futile: But It Is Slowing the Pace of EHR Adoption Nonetheless**. *J Am Med Informatics Assoc* 2009, **16**:274–281.

110. French B, Thomas LH, Baker P, Burton CR, Pennington L, Roddam H: **What can management theories offer evidence-based practice? A comparative analysis of measurement tools for organisational context.** *Implement Sci* 2009, **4**:28.

111. Fullen B, Baxter D, O’Donovan B, Doody C, Daly L, Hurley D: **Factors impacting on doctors’ management of acute low back pain: A systematic review.** *Eur J Pain* 2009:908–914.

112. Gabbay J, LeMay a: *Practice-Based Evidence for Healthcare: Clinical Mindlines*. *Volume 2010*. Routledge; 2011:1–269.

113. Gabbay J, le May A: **Evidence based guidelines or collectively constructed “mindlines?” Ethnographic study of knowledge management in primary care.** *BMJ Br Med J* 2004, **329**:1013.

114. Gagliardi AR, Wright FC: **Exploratory Evaluation of Surgical Skills Mentorship Program Design and Outcomes**. *J Contin Educ Health Prof* 2010, **30**:51–56.

115. Gagliardi AR, Wright FC, Anderson MAB, Davis D: **The role of collegial interaction in continuing professional development**. *J Contin Educ Health Prof* 2007, **27**:214–219.

116. Gagliardi AR, Wright FC, Davis D, McLeod RS, Urbach DR: **Challenges in multidisciplinary cancer care among general surgeons in Canada.** *BMC Med Inform Decis Mak* 2008, **8**:59.

117. Garg P, Lillystone D, Dossetor D, Kefford C, Chong S: **An Exploratory Survey for Understanding Perceptions, Knowledge and Educational Needs of General Practitioners (GSs) Regarding Autistic Disorders in New South Wales (NSW), Australia.** *J Clin Diagn Res* 2014, **8**:PC01–9.

118. Gask L, Rogers A, Campbell S, Sheaff R: **Beyond the limits of clinical governance? The case of mental health in English primary care.** *BMC Health Serv Res* 2008, **8**:63.

119. Gatto M.C., Armato A., Silvetti E., Salatino T., Piro A., Giunta G., Ciccaglione A., Mancone M., Fedele F.: **Obstructive sleep apnea syndrome with sinus pauses: There are indications for cardiac pacing?**. *G Ital Cardiol* 2014, **15**:e96.

120. Genuis SK, Genuis SJ: **Exploring the continuum: medical information to effective clinical practice. Paper I: the translation of knowledge into clinical practice**. *J Eval Clin Pract* 2006, **12**:49–62.

121. Genuis SJ, Schwalfenberg GK, Hiltz MN, Vaselenak SA: **Vitamin D status of clinical practice populations at higher latitudes: analysis and applications.** *Int J Environ Res Public Health* 2009, **6**:151–73.

122. Gerhardus A: **Evidence based medicine: does it make a difference? In Germany disease is treated via patients’ clinical pictures rather than by following mindlines.** *BMJ Br Med J* 2005, **330**.

123. Gething PW, Kirui VC, Alegana VA, Okiro EA, Noor AM, Snow RW: **Estimating the number of paediatric fevers associated with malaria infection presenting to Africa’s public health sector in 2007.** *PLoS Med* 2010, **7**:e1000301.

124. Gibbs L, Kealy M, Willis K, Green J, Welch N, Daly J: **What have sampling and data collection got to do with good qualitative research?**. *Aust N Z J Public Health* 2007, **31**:540–544.

125. Gildenhuys J, Lee M, Isbister GK: **Does implementation of a paediatric asthma clinical practice guideline worksheet change clinical practice?**. *Int J Emerg Med* 2009, **2**:33–9.

126. Glasziou P: **Evidence based medicine: does it make a difference?: Make it evidence informed practice with a little wisdom.** *BMJ Br Med J* 2005, **330**.

127. Goeman DP, Hogan CD, Aroni RA, Abramson MJ, Sawyer SM, Stewart K, Sanci LA, Douglass JA: **Barriers to delivering asthma care: a qualitative study of general practitioners**. *Med J Aust* 2005, **183**:457–460.

128. Gonzalez-Gonzalez AI, Dawes M, Sanchez-Mateos J, Riesgo-Fuertes R, Escortell-Mayor E, Sanz-Cuesta T, Hernandez-Fernandez T: **Information needs and information-seeking behavior of primary care physicians**. *Ann Fam Med* 2007, **5**:345–352.

129. Grant A, Sullivan F, Dowell J: **An ethnographic exploration of influences on prescribing in general practice: why is there variation in prescribing practices?**. *Implement Sci* 2013, **8**:72.

130. Greenfield D, Nugus P, Travaglia J, Braithwaite J: **Auditing an organization’s interprofessional learning and interprofessional practice: the interprofessional praxis audit framework (IPAF).** *J Interprof Care* 2010, **24**:436–49.

131. Greenhalgh J, Flynn R, Long AF, Tyson S: **Tacit and encoded knowledge in the use of standardised outcome measures in multidisciplinary team decision making: A case study of in-patient neurorehabilitation**. *Soc Sci Med* 2008, **67**:183–194.

132. Greenhalgh T: **What is this knowledge that we seek to “exchange”?**. *Milbank Q* 2010, **88**:492–9.

133. Greenhalgh T, Howick J, Maskrey N: **Evidence based medicine: a movement in crisis?**. *BMJ Br Med J* 2014, **348**.

134. Greenhalgh T, Russell J: **Evidence-based policymaking: a critique.** *Perspect Biol Med* 2009, **52**:304–18.

135. Greenhalgh T, Wieringa S: **Is it time to drop the “knowledge translation” metaphor? A critical literature review.** *J R Soc Med* 2011, **104**:501–509.

136. Gross K, Schellenberg JA, Kessy F, Pfeiffer C, Obrist B: **Antenatal care in practice: an exploratory study in antenatal care clinics in the Kilombero Valley, south-eastern Tanzania.** *BMC Pregnancy Childbirth* 2011, **11**.

137. Guo WA: **What do we use in the care of adolescent blunt abdominal solid organ injury: guidelines or “mindlines”?**. *J Surg Res* 2014, **186**:91–92.

138. Gupta S, Bhattacharyya OK, Brouwers MC, Estey EA, Harrison MB, Hernandez P, Palda VA, Boulet L-P: **Canadian Thoracic Society: Presenting a new process for clinical practice guideline production.** *Can Respir J* 2009, **16**:e62–68.

139. Gutierrez-Ibarluzea I., Ibargoyen-Roteta N., Mateos-Del Pino M., Benguria-Arrate G., Regidor-Fuentes I., Domingo-Rico C., Gonzalez-Santisteban R., Rada-Fdz De Jauregi D.: **Practice guidelines and professional mindlines. Qualitative research on sysadoas for osteoarthritis**. *BMJ Qual Saf* 2013, **22**((Gutierrez-Ibarluzea I.; Ibargoyen-Roteta N.; Mateos-Del Pino M.; Benguria-Arrate G.) Osteba (Basque Office for Health Technology Assessment), Basque Government, Vitoria- Gasteiz, Spain):A63.

140. Henry SG: **Recognizing tacit knowledge in medical epistemology**. *Theor Med Bioeth* 2006, **27**:187–213.

141. Henry SG, Zaner RM, Dittus RS: **Viewpoint: Moving beyond evidence-based medicine**. *Acad Med* 2007, **82**:292–297.

142. Hensen B, Paintain LS, Shretta R, Bruce J, Jones C, Webster J: **Taking stock: provider prescribing practices in the presence and absence of ACT stock.** *Malar J* 2011, **10**:218.

143. Hertz JT, Munishi OM, Sharp JP, Reddy EA, Crump JA: **Comparing actual and perceived causes of fever among community members in a low malaria transmission setting in northern Tanzania.** *Trop Med Int Health* 2013, **18**:1406–15.

144. Hettinga AM, Denessen E, Postma CT: **Checking the checklist: a content analysis of expert- and evidence-based case-specific checklist items**. *Med Educ* 2010, **44**:874–883.

145. Horwitz SM, Chamberlain P, Landsverk J, Mullican C: **Improving the mental health of children in child welfare through the implementation of evidence-based parenting interventions.** *Adm Policy Ment Health* 2010, **37**:27–39.

146. Howes F, Hansen E, Nelson M: **Management of hypertension in general practice A qualitative needs assessment of Australian GPs**. *Aust Fam Physician* 2012, **41**:317–+.

147. Hoy AR, Patrick H, Campbell B, Lyratzopoulos G: **Measuring the influence of colleagues on a consultant team’s use of breast conserving surgery.** *Int J Technol Assess Health Care* 2010, **26**:156–62.

148. Hoy D, Hardiker NR, McNicoll IT, Westwell P: **A Feasibility Study on Clinical Templates for the National Health Service. in Scotland**. In *Medinfo 2007 Proc 12th World Congr Heal Informatics, Pts 1 2*. *Volume 129*. Edited by Kuhn KA, Warren JR, Leong TY.; 2007:770–774.

149. Hozo I, Schell MJ, Djulbegovic B: **Decision-making when data and inferences are not conclusive: Risk-benefit and acceptable regret approach**. *Semin Hematol* 2008, **45**:150–159.

150. Hultberg J, Rudebeck CE: **Clinical Gaze in Risk-Factor Haze: Swedish GPs’ Perceptions of Prescribing Cardiovascular Preventive Drugs.** *Int J Family Med* 2012, **2012**:612572.

151. Hunt KJ: **Integrated care and the management of chronic illness: the patient’s agenda for healthy living.** 2010.

152. Ikwuobe JO, Faragher BE, Alawode G, Lalloo DG: **The impact of rapid malaria diagnostic tests upon anti-malarial sales in community pharmacies in Gwagwalada, Nigeria.** *Malar J* 2013, **12**:380.

153. Ilboudo TP, Chou Y-J, Huang N: **Assessment of providers’ referral decisions in rural Burkina Faso: a retrospective analysis of medical records.** *BMC Health Serv Res* 2012, **12**:54.

154. Ilott I: **Evidence-based practice: A critical appraisal**. *Occup Ther Int* 2012:1–6.

155. Jackson R, Baird W, Davis-Reynolds L, Smith C, Blackburn S, Allsebrook J: **The information requirements and information-seeking behaviours of health and social care professionals providing care to children with health care needs: a pilot study**. *Health Info Libr J* 2007, **24**:95–102.

156. Janes N, Sidani S, Cott C, Rappolt S: **Figuring it out in the moment: A theory of unregulated care providers’ knowledge utilization in dementia care settings**. *Worldviews Evidence-Based Nurs* 2008, **5**:13–24.

157. Jansen MWJ, van Oers HAM, Kok G, de Vries NK: **Public health: disconnections between policy, practice and research.** *Health Res Policy Syst* 2010, **8**:37.

158. Jansen M: *Mind the Gap: Collaboration between Practice, Policy and Research in Local Public Health*. Datawyse / Universitaire Pers Maastricht; 2007:297.

159. Jiwa M: **Doctors and Medical Science.** *Australas Med J* 2012, **5**:462–467.

160. Jiwa M, Deas K, Ross J, Shaw T, Wilcox H, Spilsbury K: **An inclusive approach to raising standards in general practice: working with a “community of practice” in Western Australia.** *BMC Med Res Methodol* 2009, **9**:13.

161. Jiwa M, Gordon M, Arnet H, Ee H, Bulsara M, Colwell B: **Referring patients to specialists: a structured vignette survey of Australian and British GPs.** *BMC Fam Pract* 2008, **9**:2.

162. Jiwa M, Saunders C: **Fast track referral for cancer - Has not improved patient outcomes in the UK**. *BMJ Br Med J* 2007, **335**:267–268.

163. Johansson EW, Gething PW, Hildenwall H, Mappin B, Petzold M, Peterson SS, Selling KE: **Diagnostic testing of pediatric fevers: meta-analysis of 13 national surveys assessing influences of malaria endemicity and source of care on test uptake for febrile children under five years.** *PLoS One* 2014, **9**:e95483.

164. Johansson ME, Pilhammar E, Khalaf A, Willman A: **Registered nurses’ adherence to clinical guidelines regarding peripheral venous catheters: A structured observational study**. *Worldviews Evidence-Based Nurs* 2008:148–159.

165. Jonas WB: **Scientific evidence and medical practice: The “Drunkard’s Walk”.** *Arch Intern Med* 2009:649–650.

166. Jones COH, Wasunna B, Sudoi R, Githinji S, Snow RW, Zurovac D: **“Even if you know everything you can forget”: health worker perceptions of mobile phone text-messaging to improve malaria case-management in Kenya.** *PLoS One* 2012, **7**:e38636.

167. Jones R: **How The Light Gets In: Practice-based Evidence for Healthcare Clinical Mindlines John Gabbay and Andree le May**. *Br J Gen Pract* 2014, **64**:250–250.

168. Jones T: **Information on common conditions: the development and implementation of a structured web-based resource to enable access to clinical information for clinicians in primary care.** *Educ Prim Care* 2007, **18**(July 2007):516–522.

169. Joseph D, Kabanywanyi AM, Hulser R, Premji Z, Minzi OMS, Mugittu K: **Exploration of in vivo efficacy of artemether-lumefantrine against uncomplicated Plasmodium falciparum malaria in under fives in Tabora region, Tanzania.** *Malar J* 2013, **12**:60.

170. Keating NL, Ayanian JZ, Cleary PD, Marsden P V: **Factors affecting influential discussions among physicians: a social network analysis of a primary care practice.** *J Gen Intern Med* 2007, **22**:794–798.

171. Kernick D: **Developing a research agenda in headache service delivery: proceed with caution**. *Cephalalgia* 2007, **27**:289–293.

172. Kernick D: **Wanted - new methodologies for health service research. Is complexity theory the answer?**. *Fam Pract* 2006, **23**:385–390.

173. Kernick DP: **Evidence based medicine: does it make a difference?: Management of complex systems needs new approaches.** *BMJ Br Med J* 2005, **330**:92–93.

174. Kimble C, Bourdon I: **Some success factors for the communal management of knowledge**. *Int J Inf Manage* 2008, **28**:461–467.

175. Kipanga PN, Omondi D, Mireji PO, Sawa P, Masiga DK, Villinger J: **High-resolution melting analysis reveals low Plasmodium parasitaemia infections among microscopically negative febrile patients in western Kenya.** *Malar J* 2014, **13**:429.

176. Kislov R, Walshe K, Harvey G: **Managing boundaries in primary care service improvement: a developmental approach to communities of practice.** *Implement Sci* 2012, **7**:97.

177. Knowles S, Mcinnes E, Elliott D, Hardy J, Middleton S: **Evaluation of the implementation of a bowel management protocol in intensive care: Effect on clinician practices and patient outcomes**. *J Clin Nurs* 2014:716–730.

178. Kosteniuk JG, Morgan DG, D’Arcy CK: **Use and perceptions of information among family physicians: sources considered accessible, relevant, and reliable.** *J Med Libr Assoc* 2013, **101**:32–7.

179. Kothari AR, Bickford JJ, Edwards N, Dobbins MJ, Meyer M: **Uncovering tacit knowledge: a pilot study to broaden the concept of knowledge in knowledge translation.** *BMC Health Serv Res* 2011, **11**:198.

180. Kothari A, Rudman D, Dobbins M, Rouse M, Sibbald S, Edwards N: **The use of tacit and explicit knowledge in public health: a qualitative study.** *Implement Sci* 2012, **7**:20.

181. Kyabayinze DJ, Asiimwe C, Nakanjako D, Nabakooza J, Counihan H, Tibenderana JK: **Use of RDTs to improve malaria diagnosis and fever case management at primary health care facilities in Uganda.** *Malar J* 2010, **9**:200.

182. Kyratsis Y, Ahmad R, Hatzaras K, Iwami M, Holmes A: **Making sense of evidence in management decisions: the role of research-based knowledge on innovation adoption and implementation in health care**. *Heal Serv Deliv Res* 2014, **2**:1–192.

183. Lacasta Tintorer D, Flayeh Beneyto S, Alzaga Reig X, Mundet Tuduri X, De la Fuente JA, Manresa JM, Torán Monserrat P, Saigí Rubió F: **Impact of the implementation of an online network support tool among clinicians of Primary Health Care and Specialists: ECOPIH Project.** *BMC Fam Pract* 2013, **14**:146.

184. Lange S, Mwisongo A, Maestad O: **Why don’t clinicians adhere more consistently to guidelines for the Integrated Management of Childhood Illness (IMCI)?**. *Soc Sci Med* 2014, **104**:56–63.

185. Le May A: *Communities of Practice in Health and Social Care*. John Wiley & Sons; 2008:144.

186. Le May A, Holmes S: *Introduction to Nursing Research: Developing Research Awareness*. CRC Press; 2012:149.

187. Leasure a. R, Stirlen J, Thompson C: **Barriers and Facilitators to the Use of Evidence-Based Best Practices**. *Dimens Crit Care Nurs* 2008, **27**:74–82.

188. Lessard C, Contandriopoulos A-PA-P, Beaulieu M-D: **The role of economic evaluation in the decision-making process of family physicians: design and methods of a qualitative embedded multiple-case study.** *BMC Fam Pract* 2009, **10**:15.

189. Levine D, Bleakley A: **Maximising medicine through aphorisms**. *Med Educ* 2012:153–162.

190. Lewith G, Barlow F, Eyles C, Flower A, Hall S, Hopwood V, Walker J: **The context and meaning of placebos for complementary medicine.** *Forsch Komplementmed* 2009, **16**:404–12.

191. Lewith G, Brien S, Barlow F, Eyles C, Flower A, Hall S, Hill C, Hopwood V: **The meaning of evidence: can practitioners be researchers?**. *Forsch Komplementmed* 2009, **16**:343–7.

192. Li LC, Grimshaw JM, Nielsen C, Judd M, Coyte PC, Graham ID: **Evolution of Wenger’s concept of community of practice**. *Implement Sci* 2009, **4**:11.

193. Lockyer J, Armson H, Chesluk B, Dornan T, Holmboe E, Loney E, Mann K, Sargeant J: **Feedback data sources that inform physician self-assessment**. *Med Teach* 2011, **33**:e113–e120.

194. Lofgren A, Silen C, Alexanderson K: **How physicians have learned to handle sickness-certification cases**. *Scand J Public Health* 2011, **39**:245–254.

195. Lomas J: **The in-between world of knowledge brokering.** *BMJ* 2007, **334**:129–32.

196. Loughlin M, Lewith G, Falkenberg T: **Science, Practice and Mythology: A Definition and Examination of the Implications of Scientism in Medicine**. *Heal Care Anal* 2013, **21**:130–145.

197. Louro Gonzalez A, Fernandez Obanza E, Fernandez Lopez E, Vazquez Millan P, Villegas Gonzalez L, Casariego Vales E: **Analysis of the doubts of primary care doctors**. *Aten Primaria* 2009, **41**:592–597.

198. Lucchiari C, Pravettoni G: **Cognitive balanced model: A conceptual scheme of diagnostic decision making**. *J Eval Clin Pract* 2012:82–88.

199. Luker K, Orr J, McHugh G: *Health Visiting: A Rediscovery*. John Wiley & Sons; 2012:269.

200. Lutfey KE, Link CL, Grant RW, Marceau LD, McKinlay JB: **Is certainty more important than diagnosis for understanding race and gender disparities?: an experiment using coronary heart disease and depression case vignettes.** *Health Policy* 2009, **89**:279–87.

201. Malhi GS, Adams D: **Are guidelines in need of CPR? The development of clinical practice recommendations (CPR).** *Acta Psychiatr Scand Suppl* 2009, **119**:5–7.

202. Malterud K: **The social construction of clinical knowledge - the context of culture and discourse. Commentary on Tonelli (2006), Integrating evidence into clinical practice: an alternative to evidence-based approaches. Journal of Evaluation in Clinical Practice 12, 24**. *J Eval Clin Pract* 2006, **12**:292–295.

203. Martinez-Valverde S, Castro-Rios A, Perez-Cuevas R, Klunder-Klunder M, Salinas-Escudero G, Reyes-Morales H: **Effectiveness of a medical education intervention to treat hypertension in primary care**. *J Eval Clin Pract* 2012, **18**:420–425.

204. Martins JSJS, Zwi AB, Hobday K, Bonaparte F, Kelly PM: **The implementation of a new Malaria Treatment Protocol in Timor-Leste: challenges and constraints.** *Health Policy Plan* 2012, **27**:677–86.

205. Mascia D, Cicchetti A, Damiani G: **“Us and Them”: a social network analysis of physicians’ professional networks and their attitudes towards EBM.** *BMC Health Serv Res* 2013, **13**:429.

206. Mascia D, Dandi R, Di Vincenzo F: **Professional networks and EBM use: A study of inter-physician interaction across levels of care**. *Health Policy (New York)* 2014, **118**:24–36.

207. Maskrey N, Underhill J, Hutchinson a., Shaughnessy a., Slawson D: **Getting a better grip on research: A simple system that works**. *InnovAiT* 2009:739–749.

208. Maskrey N, Underhill J, Hutchinson a., Shaughnessy a., Slawson D: **Getting a better grip on research: A simple system that works**. *InnovAiT* 2009:739–749.

209. Maskrey N, Underhill J, Hutchinson A, Shaughnessy A, Slawson D: **Getting a better grip on research: the maze of the most busy life**. *InnovAiT* 2010:172–9.

210. Maskrey N, T: **Getting a better grip on research: the fate of those who ignore history**. *InnovAiT* 2009, **2**:619–625.

211. Mason VL, Shaw A, Wiles NJ, Mulligan J, Peters TJ, Sharp D, Lewis G: **GPs’ experiences of primary care mental health research: a qualitative study of the barriers to recruitment**. *Fam Pract* 2007, **24**:518–525.

212. May L, Gudger G, Armstrong P, Brooks G, Hinds P, Bhat R, Moran GJ, Schwartz L, Cosgrove SE, Klein EY, Rothman RE, Rand C: **Multisite exploration of clinical decision making for antibiotic use by emergency medicine providers using quantitative and qualitative methods.** *Infect Control Hosp Epidemiol* 2014:1114–25.

213. Mays NB: **The Australian primary health care research institute: rising to the challenge of applying knowledge from research to Australian policy**. *Med J Aust* 2008, **188**:S44–S45.

214. Mbonye AK, Ndyomugyenyi R, Turinde A, Magnussen P, Clarke S, Chandler C: **The feasibility of introducing rapid diagnostic tests for malaria in drug shops in Uganda.** *Malar J* 2010, **9**:367.

215. McCaughan D: **Primary care practitioners based everyday practice on internalised tacit guidelines derived through social interactions with trusted colleagues.** *Evid Based Nurs* 2005:94.

216. McCrate F, Godwin M, Murphy L: **Attainment of Canadian Diabetes Association recommended targets in patients with type 2 diabetes: A study of primary care practices in St John’s, Nfld.** *Can Fam Physician* 2010, **56**:e13–19.

217. McCurtin A, Roddam H: **Evidence-based practice: SLTs under siege or opportunity for growth? The use and nature of research evidence in the profession**. *Int J Lang Commun Disord* 2012, **47**:11–26.

218. McDonald R, Waring J, Harrison S: **Rules, safety and the narrativisation of identity: a hospital operating theatre case study**. *Sociol Health Illn* 2006, **28**:178–202.

219. McKibbon KA, Lokker C, Wilczynski NL, Ciliska D, Dobbins M, Davis DA, Haynes RB, Straus SE: **A cross-sectional study of the number and frequency of terms used to refer to knowledge translation in a body of health literature in 2006: a Tower of Babel?**. *Implement Sci* 2010, **5**:16.

220. Meagher-Stewart D, Solberg SM, Warner G, MacDonald J-A, McPherson C, Seaman P: **Understanding the Role of Communities of Practice in Evidence-Informed Decision Making in Public Health**. *Qual Health Res* 2012, **22**:723–739.

221. Mikhail AFW, Leslie TJ, Mayan MI, Zekria R, Mohammad N, Hasanzai MA, Safi N, Whitty CJM, Rowland M: **Field trial of three different Plasmodium vivax-detecting rapid diagnostic tests with and without evaporative cool box storage in Afghanistan.** *Malar J* 2011, **10**.

222. Moffat M, Cleland J, van der Molen T, Price D: **Poor communication may impair optimal asthma care: a qualitative study**. *Fam Pract* 2007, **24**:65–70.

223. Moja L, Banzi R: **Navigators for medicine: evolution of online point-of-care evidence-based services.** *Int J Clin Pract* 2011, **65**:6–11.

224. Moja L, Liberati EG, Galuppo L, Gorli M, Maraldi M, Nanni O, Rigon G, Ruggieri P, Ruggiero F, Scaratti G, Vaona A, Kwag KH: **Barriers and facilitators to the uptake of computerized clinical decision support systems in specialty hospitals: protocol for a qualitative cross-sectional study**. *Implement Sci* 2014, **9**:105.

225. Moon AM, Biggs HM, Rubach MP, Crump JA, Maro VP, Saganda W, Reddy EA: **Evaluation of In-Hospital Management for Febrile Illness in Northern Tanzania before and after 2010 World Health Organization Guidelines for the Treatment of Malaria.** *PLoS One* 2014, **9**.

226. Morris ZS, Clarkson PJ: **Does social marketing provide a framework for changing healthcare practice?**. *Health Policy (New York)* 2009, **91**:135–141.

227. Mosha JF, Conteh L, Tediosi F, Gesase S, Bruce J, Chandramohan D, Gosling R: **Cost Implications of Improving Malaria Diagnosis: Findings from North-Eastern Tanzania.** *PLoS One* 2010, **5**.

228. Mtove G, Hendriksen ICE, Amos B, Mrema H, Mandia V, Manjurano A, Muro F, Sykes A, Hildenwall H, Whitty CJM, Reyburn H: **Treatment guided by rapid diagnostic tests for malaria in Tanzanian children: safety and alternative bacterial diagnoses.** *Malar J* 2011, **10**.

229. Mubi M, Kakoko D, Ngasala B, Premji Z, Peterson S, Bjorkman A, Martensson A: **Malaria diagnosis and treatment practices following introduction of rapid diagnostic tests in Kibaha District, Coast Region, Tanzania.** *Malar J* 2013, **12**.

230. Mwanziva C, Shekalaghe S, Ndaro A, Mengerink B, Megiroo S, Mosha F, Sauerwein R, Drakeley C, Gosling R, Bousema T: **Overuse of artemisinin-combination therapy in Mto wa Mbu (river of mosquitoes), an area misinterpreted as high endemic for malaria.** *Malar J* 2008, **7**:232.

231. Nankabirwa J, Zurovac D, Njogu JN, Rwakimari JB, Counihan H, Snow RW, Tibenderana JK: **Malaria misdiagnosis in Uganda--implications for policy change.** *Malar J* 2009, **8**:66.

232. Nausheen B, Gidron Y, Peveler RC, Bruce L, Verrill C: **The relationship between social support and disease markers in colorectal cancer**. In *Psychooncology*. *Volume 15*; 2006:S1–S478.

233. Nicolini D, Powell J, Conville P, Martinez-Solano L: **Managing knowledge in the healthcare sector. A review**. *Int J Manag Rev* 2008, **10**:245–263.

234. Nieri M, Mauro S: **Continuing professional development of dental practitioners in Prato, Italy**. *J Dent Educ* 2008, **72**:616–625.

235. Nishio C, Hajiro T, Nagao T, Ito M, Sakaguchi C, Nakano Y: **Specialists play a vital role in general practitioners’ prescription behavior: A qualitative study of asthma care in Japan**. *J Asthma* 2008, **45**:339–342.

236. Nissen N, Weidenhammer W, Schunder-Tatzber S, Johannessen H: **Public health ethics for complementary and alternative medicine**. *Eur J Integr Med* 2013, **5**:62–67.

237. Njozi M, Amuri M, Selemani M, Masanja I, Kigahe B, Khatib R, Kajungu D, Abdula S, Dodoo AN: **Predictors of antibiotics co-prescription with antimalarials for patients presenting with fever in rural Tanzania.** *BMC Public Health* 2013, **13**.

238. Nutley SM, Walter I DH: *Using Evidence. How Research Can Inform Public Services.* Policy Press; 2007:363.

239. Nutley S: **Debate: Are we all co-producers of research now?**. *Public Money Manag* 2010, **30**:263–265.

240. Nzinga J, Mbindyo P, Mbaabu L, Warira A, English M: **Documenting the experiences of health workers expected to implement guidelines during an intervention study in Kenyan hospitals.** *Implement Sci* 2009, **4**.

241. Oborn E, Dawson S: **Knowledge and practice in multidisciplinary teams: Struggle, accommodation and privilege**. *Hum Relations* 2010, **63**:1835–1857.

242. Oduro-Mensah E, Kwamie A, Antwi E, Amissah Bamfo S, Bainson HM, Marfo B, Coleman MA, Grobbee DE, Agyepong IA: **Care decision making of frontline providers of maternal and newborn health services in the greater Accra region of Ghana.** *PLoS One* 2013, **8**:e55610.

243. Okebe JU, Walther B, Bojang K, Drammeh S, Schellenberg D, Conway DJ, Walther M: **Prescribing practice for malaria following introduction of artemether-lumefantrine in an urban area with declining endemicity in West Africa.** *Malar J* 2010, **9**.

244. Onwujekwe O, Uzochukwu B, Dike N, Uguru N, Nwobi E, Shu E: **Malaria treatment perceptions, practices and influences on provider behaviour: comparing hospitals and non-hospitals in south-east Nigeria.** *Malar J* 2009, **8**.

245. Paintain LS, Willey B, Kedenge S, Sharkey A, Kim J, Buj V, Webster J, Schellenberg D, Ngongo N: **Community Health Workers and Stand-Alone or Integrated Case Management of Malaria: A Systematic Literature Review.** *Am J Trop Med Hyg* 2014, **91**:461–470.

246. Parboosingh IJ, Reed VA, Palmer JC, Bernstein HH: **Enhancing Practice Improvement by Facilitating Practitioner Interactivity: New Roles for Providers of Continuing Medical Education**. *J Contin Educ Health Prof* 2011, **31**:122–127.

247. Parker M: **False dichotomies: EBM, clinical freedom, and the art of medicine**. *Med Humanit* 2005, **31**:23–30.

248. Peiris D, Usherwood T, Weeramanthri T, Cass A, Patel A: **New tools for an old trade: a socio-technical appraisal of how electronic decision support is used by primary care practitioners.** *Sociol Health Illn* 2011, **33**:1002–18.

249. Pell C, Meñaca A, Afrah NA, Manda-Taylor L, Chatio S, Were F, Hodgson A, Hamel MJ, Kalilani L, Tagbor H, Pool R: **Prevention and management of malaria during pregnancy: findings from a comparative qualitative study in Ghana, Kenya and Malawi.** *Malar J* 2013, **12**:427.

250. Pell C, Meñaca A, Chatio S, Hodgson A, Tagbor H, Pool R: **The acceptability of intermittent screening and treatment versus intermittent preventive treatment during pregnancy: results from a qualitative study in Northern Ghana.** *Malar J* 2014, **13**:432.

251. Petto P, Hinds PS: **Out With the Old, In With the New: New Findings Replacing Previous Care Practices**. *Cancer Nurs* 2009, **32**:343–344.

252. Petursson P: **GPs’ reasons for "non-pharmacological'' prescribing of antibiotics - A phenomenological study**. *Scand J Prim Health Care* 2005, **23**:120–125.

253. Phipps DL, Parker D, Pals EJM, Meakin GH, Nsoedo C, Beatty PCW: **Identifying violation-provoking conditions in a healthcare setting.** *Ergonomics* 2008, **51**:1625–42.

254. Pi SD, Bennett C, Fitzgerald L, Fischer M, Ledger J, Mcculloch J, Mcgivern G: *Health Care Managers ’ Access and Use of Management Research*. 2013.

255. Powers JH: **Practice Guidelines Belief, Criticism, and Probability**. *Arch Intern Med* 2011, **171**:15–17.

256. Pulford J, Mueller I, Siba PM, Hetzel MW: **Malaria case management in Papua New Guinea prior to the introduction of a revised treatment protocol.** *Malar J* 2012, **11**.

257. Qaseem A, Forland F, Macbeth F, Ollenschlaeger G, Phillips S, van der Wees P: **Guidelines International Network: Toward International Standards for Clinical Practice Guidelines**. *Ann Intern Med* 2012, **156**:525–U103.

258. Rakel D, Rakel RE: *Textbook of Family Medicine*. Elsevier Health Sciences; 2011:1192.

259. Randell R, Mitchell N, Thompson C, McCaughan D, Dowding D: **Supporting nurse decision making in primary care: exploring use of and attitude to decision tools**. *Health Informatics J* 2009, **15**:5–16.

260. Ranmuthugala G, Plumb JJ, Cunningham FC, Georgiou A, Westbrook JI, Braithwaite J: **How and why are communities of practice established in the healthcare sector? A systematic review of the literature.** *BMC Health Serv Res* 2011, **11**:273.

261. Rao VB, Schellenberg D, Ghani AC: **The potential impact of improving appropriate treatment for fever on malaria and non-malarial febrile illness management in under-5s: a decision-tree modelling approach.** *PLoS One* 2013, **8**:e69654.

262. Rapley T: **Distributed decision making: the anatomy of decisions-in-action**. *Sociol Health Illn* 2008, **30**:429–444.

263. Reach G: **Clinical inertia, uncertainty and individualized guidelines**. *Diabetes Metab* 2014, **40**:241–245.

264. Reeve J, Dowrick CF, Freeman GK, Gunn J, Mair F, May C, Mercer S, Palmer V, Howe A, Irving G, Shiner A, Watson J: **Examining the practice of generalist expertise: a qualitative study identifying constraints and solutions.** *JRSM Short Rep* 2013, **4**.

265. Reeve J, Irving G, Dowrick CF: **Can generalism help revive the primary healthcare vision?**. *J R Soc Med* 2011, **104**.

266. Repullo Labrador JR: **Identification and Modulation of the Systematic Irrational Behaviour in Medicine and Public Health**. *Rev Esp Salud Publica* 2009, **83**:43–57.

267. Reynolds J, DiLiberto D, Mangham-Jefferies L, Ansah EK, Lal S, Mbakilwa H, Bruxvoort K, Webster J, Vestergaard LS, Yeung S, Leslie T, Hutchinson E, Reyburn H, Lalloo DG, Schellenberg D, Cundill B, Staedke SG, Wiseman V, Goodman C, Chandler CIR: **The practice of “doing” evaluation: lessons learned from nine complex intervention trials in action.** *Implement Sci* 2014, **9**.

268. Rieger K, Schultz ASH: **Exploring arts-based knowledge translation: sharing research findings through performing the patterns, rehearsing the results, staging the synthesis.** *Worldviews Evid Based Nurs* 2014:133–9.

269. Robertson J, Moxey AJ, Newby D a, Gillies MB, Williamson M, Pearson S-A: **Electronic information and clinical decision support for prescribing: state of play in Australian general practice.** *Fam Pract* 2011, **28**:93–101.

270. Robertson L, Graham F, Anderson J: **What actually informs practice: occupational therapists’ views of evidence**. *Br J Occup Ther* 2013, **76**:317–324.

271. Rollnick S, Butler CC, McCambridge J, Kinnersley P, Elwyn G, Resnicow K: **Consultations about changing behaviour**. *BMJ Br Med J* 2005, **331**:961–963.

272. Rowe AK, de Savigny D, Lanata CF, Victora CG: **How can we achieve and maintain high-quality performance of health workers in low-resource settings?**. *Lancet* 2005, **366**:1026–1035.

273. Rowe R, McDaid D: **Implementation: the need for a contextual approach to the implementation of musculoskeletal guidelines**. *Best Pract Res Clin Rheumatol* 2007, **21**:205–219.

274. Runciman B: **Is it time for consultants to join trainees in working night shifts? Yes.** *Emerg Med Australas* 2014, **26**:506–507.

275. Rushmer RK, Hunter DJ, Steven A: **Using interactive workshops to prompt knowledge exchange: a realist evaluation of a knowledge to action initiative**. *Public Health* 2014, **128**:552–560.

276. Russell G, Advocat J, Geneau R, Farrell B, Thille P, Ward N, Evans S: **Examining organizational change in primary care practices: experiences from using ethnographic methods**. *Fam Pract* 2012, **29**:455–461.

277. Rycroft-Malone J, Seers K, Chandler J, Hawkes CA, Crichton N, Allen C, Bullock I, Strunin L: **The role of evidence, context, and facilitation in an implementation trial: implications for the development of the PARIHS framework.** *Implement Sci* 2013, **8**:28.

278. Rycroft-Malone J, Wilkinson JE, Burton CR, Andrews G, Ariss S, Baker R, Dopson S, Graham I, Harvey G, Martin G, McCormack BG, Staniszewska S, Thompson C: **Implementing health research through academic and clinical partnerships: a realistic evaluation of the Collaborations for Leadership in Applied Health Research and Care (CLAHRC).** *Implement Sci* 2011, **6**.

279. Sargeant J, Curran V, Allen M, Jarvis-Selinger S, Ho K: **Facilitating interpersonal interaction and learning online: Linking theory and practice**. *J Contin Educ Health Prof* 2006, **26**:128–136.

280. Sargeant J, Hurley KF, Duffy J, Sketris I, Sinclair D, Ducharme J: **Lost in Translation or Just Lost?**. *Ann Emerg Med* 2008:575–576.

281. Sarrassat S, Lalou R, Cissé M, Le Hesran J-Y: **Management of uncomplicated malaria in children under 13 years of age at a district hospital in Senegal: from official guidelines to usual practices.** *Malar J* 2011, **10**:285.

282. Scott I: **The evolving science of translating research evidence into clinical practice.** *Evid Based Med* 2007:4–7.

283. Scott I a: **Errors in clinical reasoning: causes and remedial strategies.** *BMJ* 2009:b1860.

284. Scott K, McSherry R: **Evidence-based nursing: clarifying the concepts for nurses in practice**. *J Clin Nurs* 2009, **18**:1085–1095.

285. Scotti LA, Greenberg PB, Phillips PA: **Strengthening the scientific approach to clinical practice in the new physician training programme**. *Intern Med J* 2008, **38**:384–387.

286. Sharma KK, Gupta R, Agrawal A, Roy S, Kasliwal A, Bana A, Tongia RK, Deedwania PC: **Low use of statins and other coronary secondary prevention therapies in primary and secondary care in India.** *Vasc Health Risk Manag* 2009, **5**:1007–1014.

287. Sharma KK, Mathur M, Gupta R, Guptha S, Roy S, Khedar RS, Gupta N, Gupta R: **Epidemiology of cardioprotective pharmacological agent use in stable coronary heart disease.** *Indian Heart J* 2013, **65**:250–5.

288. Siddiqi N, Young J, Cheater FM, Harding RA: **Educating staff working in long-term care about delirium: The Trojan horse for improving quality of care?**. *J Psychosom Res* 2008, **65**:261–266.

289. Sievers AC, Lewey J, Musafiri P, Franke MF, Bucyibaruta BJ, Stulac SN, Rich ML, Karema C, Daily JP: **Reduced paediatric hospitalizations for malaria and febrile illness patterns following implementation of community-based malaria control programme in rural Rwanda.** *Malar J* 2008, **7**:167.

290. Silversides A: **Patient-safety reforms inhibited by systemic impediments.** *CMAJ* 2008, **179**:1253–5.

291. Skånér Y, Nilsson GH, Arrelöv B, Lindholm C, Hinas E, Wilteus ALL, Alexanderson K, Skaner Y, Arrelov B: **Use and usefulness of guidelines for sickness certification: results from a national survey of all general practitioners in Sweden.** *BMJ Open* 2011, **1**:e000303.

292. Smart A: **A multi-dimensional model of clinical utility**. *Int J Qual Heal Care* 2006, **18**:377–382.

293. Smith a., Alderson P: **Guidelines in anaesthesia: Support or constraint?**. *Br J Anaesth* 2012:1–4.

294. Smith J: **From optimism to hubris.** *BMJ Br Med J* 2004, **329**.

295. Soubhi H, Bayliss EA, Fortin M, Hudon C, van den Akker M, Thivierge R, Posel N, Fleiszer D: **Learning and caring in communities of practice: using relationships and collective learning to improve primary care for patients with multimorbidity.** *Ann Fam Med* 2010, **8**:170–7.

296. Standing M: **Clinical decision-making skills on the developmental journey from student to Registered Nurse: a longitudinal inquiry**. *J Adv Nurs* 2007, **60**:257–269.

297. Starcevic V: **The reappraisal of benzodiazepines in the treatment of anxiety and related disorders.** *Expert Rev Neurother* 2014, **14**:1275–1286.

298. Steinhardt LC, Chinkhumba J, Wolkon A, Luka M, Luhanga M, Sande J, Oyugi J, Ali D, Mathanga D, Skarbinski J: **Patient-, health worker-, and health facility-level determinants of correct malaria case management at publicly funded health facilities in Malawi: results from a nationally representative health facility survey.** *Malar J* 2014, **13**:64.

299. Struck R, Baumgarten G, Wittmann M: **Cost-efficiency of knowledge creation: randomized controlled trials vs. observational studies.** *Curr Opin Anaesthesiol* 2014, **27**:190–4.

300. Suchman AL: **Organizations as Machines, Organizations as Conversations Two Core Metaphors and Their Consequences**. *Med Care* 2011, **49**:S43–S48.

301. Sudbery J: *Social Work Skills: A Practice Handbook*. *Volume 17*. McGraw-Hill International; 2003:193–194.

302. Suurmond J, Rupp I, Seeleman C, Goosen S, Stronks K: **The first contacts between healthcare providers and newly-arrived asylum seekers: a qualitative study about which issues need to be addressed**. *Public Health* 2013, **127**:668–673.

303. Swayne J: **Complementary/alternative medicine: engulfed by postmodernism, anti-science and regressive thinking COMMENTARY**. *Br J Gen Pract* 2009, **59**:301.

304. Swayne J: **The problem with science-the context and process of care: an excerpt from remodelling medicine.** *Glob Adv Health Med* 2012, **1**:78–87.

305. Swennen MHJ, Van Der Heijden GJMG, Blijham GH, Kalkman CJ: **Career stage and work setting create different barriers for evidence-based medicine**. *J Eval Clin Pract* 2011, **17**:775–785.

306. Swennen MHJ, van der Heijden GJMG, Boeije HR, van Rheenen N, Verheul FJM, van der Graaf Y, Kalkman CJ: **Doctors’ Perceptions and Use of Evidence-Based Medicine: A Systematic Review and Thematic Synthesis of Qualitative Studies**. *Acad Med* 2013, **88**:1384–1396.

307. Swinglehurst D: **Evidence-based guidelines: The theory and the practice**. *Evidence-Based Healthc Public Heal* 2005, **9**:308–314.

308. Tavazzi L: **Do we need clinical registries?**. *Eur Heart J* 2014, **35**:7–9.

309. te Pas E, Van Dijk N, Bartelink MEL, Wieringa-De Waard M: **Factors influencing the EBM behaviour of GP trainers: A mixed method study**. *Med Teach* 2013, **35**:E990–E997.

310. Thomas LH, Watkins CL, French B, Sutton C, Forshaw D, Cheater F, Roe B, Leathley MJ, Burton C, McColl E, Booth J: **Study protocol: ICONS: Identifying continence options after stroke: A randomised trial.** *Trials* 2011, **12**.

311. Thompson C, Aitken L, Doran D, Dowding D: **An agenda for clinical decision making and judgement in nursing research and education**. *Int J Nurs Stud* 2013, **50**:1720–1726.

312. Thompson C, Bucknall T, Estabrookes CA, Hutchinson A, Fraser K, de Vos R, Binnecade J, Barrat G, Saunders J: **Nurses’ critical event risk assessments: a judgement analysis**. *J Clin Nurs* 2009, **18**:601–612.

313. Thompson C, Spilsbury K, Dowding D, Pattenden J, Brownlow R: **Do heart failure specialist nurses think differently when faced with “hard” or “easy” decisions: a judgement analysis**. *J Clin Nurs* 2008, **17**:2174–2184.

314. Tolson D, Lowndes A, Booth J, Schofield I, Wales A: **The potential of communities of practice to promote evidence-informed practice within nursing homes.** *J Am Med Dir Assoc* 2011, **12**:169–73.

315. Tolson D, McIntosh J, Loftus L, Cormie P: **Developing a managed clinical network in palliative care: a realistic evaluation**. *Int J Nurs Stud* 2007, **44**:183–195.

316. Treasure W: **First do no harm: dropping in on members of the team, encouraging and thanking them.** *Br J Gen Pract* 2013, **63**:45.

317. Trevithick P: **Revisiting the knowledge base of social work: A framework for practice**. *Br J Soc Work* 2008, **38**:1212–1237.

318. Tsai KY: **Evidence-based medicine - Do we use guidelines or mindlines?**. *Arch Dermatol* 2005, **141**:773–774.

319. Unwin J, Peters D: **Gatekeepers and the Gateway--a mixed-methods inquiry into practitioners’ referral behaviour to the Gateway Clinic.** *Acupunct Med* 2009:21–25.

320. Upton D, Upton P: **Knowledge and use of evidence-based practice of GPs and hospital doctors**. *J Eval Clin Pract* 2006, **12**:376–384.

321. Urquhart R, Cornelissen E, Lal S, Colquhoun H, Klein G, Richmond S, Witteman HO: **A Community of Practice for Knowledge Translation Trainees: An Innovative Approach for Learning and Collaboration**. *J Contin Educ Health Prof* 2013, **33**:274–281.

322. Urquhart R, Porter GA, Grunfeld E, Sargeant J: **Exploring the interpersonal-, organization-, and system-level factors that influence the implementation and use of an innovation-synoptic reporting-in cancer care.** *Implement Sci* 2012, **7**:12.

323. Uzochukwu BSC, Obikeze EN, Onwujekwe OE, Onoka CA, Griffiths UK: **Cost-effectiveness analysis of rapid diagnostic test, microscopy and syndromic approach in the diagnosis of malaria in Nigeria: implications for scaling-up deployment of ACT.** *Malar J* 2009, **8**:265.

324. Uzochukwu BSC, Onwujekwe OE, Uguru NP, Ughasoro MD, Ezeoke OP: **Willingness to pay for rapid diagnostic tests for the diagnosis and treatment of malaria in southeast Nigeria: ex post and ex ante.** *Int J Equity Health* 2010, **9**.

325. Vasileiou K, Barnett J, Young T: **The Production and Use of Evidence in Health Care Service Innovation: A Qualitative Study**. *Eval Health Prof* 2013, **36**:93–105.

326. Visram S, Goodall D, Steven A: **Exploring conceptualizations of knowledge translation, transfer and exchange across public health in one UK region: a qualitative mapping study.** *Public Health* 2014, **128**:497–503.

327. Voogdt-Pruis HR, Beusmans GHMI, Gorgels APM, van Ree JW: **Experiences of doctors and nurses implementing nurse-delivered cardiovascular prevention in primary care: a qualitative study**. *J Adv Nurs* 2011, **67**:1758–1766.

328. Wagner CG: **Thinking globally, acting locally, living personally**. *Ann Emerg Med* 2005:61–63.

329. Walach H, Falkenberg T, F?nneb? V, Lewith G, Jonas WB: **Circular instead of hierarchical: methodological principles for the evaluation of complex interventions.** *BMC Med Res Methodol* 2006, **6**.

330. Walczak S, Mann R: **Utilization and Perceived Benefit for Diverse Users of Communities of Practice in a Healthcare Organization**. *J Organ End User Comput* 2010, **22**:24–50.

331. Wensley R: *Effective Management in Practice: Analytical Insights and Critical Questions*. SAGE Publications; 2013:248.

332. Whitty CJM, Chandler C, Ansah E, Leslie T, Staedke SG: **Deployment of ACT antimalarials for treatment of malaria: challenges and opportunities.** *Malar J* 2008, **7**(Suppl 1).

333. Williams B, Skinner J, Dowell J, Roberts R, Crombie I, Davis J: **General practitioners’ reasons for the failure of a randomized controlled trial (The TIGER Trial) to implement epilepsy guidelines in primary care.** *Epilepsia* 2007, **48**:1275–82.

334. Willis RK: *Evidence-Based Healthcare in Context: Critical Social Science Perspectives*. *Volume 36*. Ashgate Publishing, Ltd.; 2012:358.

335. Wiseman V, Ogochukwu E, Emmanuel N, Lindsay J M, Bonnie C, Jane E, Eloka U, Benjamin U, Obinna O: **A cost-effectiveness analysis of provider and community interventions to improve the treatment of uncomplicated malaria in Nigeria: study protocol for a randomized controlled trial.** *Trials* 2012, **13**.

336. Wright FC, Simunovic M, Coates A, Fitch M: **Quality initiative in rectal cancer strategy: A qualitative study of participating surgeons**. *J Am Coll Surg* 2006, **203**:795–802.

337. Wye L, Shaw A, Sharp D: **Patient choice and evidence based decisions: the case of complementary therapies**. *Heal Expect* 2009, **12**:321–330.

338. Yamazaki H, Slingsby BT, Takahashi M, Hayashi Y, Sugimori H, Nakayama T: **Characteristics of qualitative studies in influential journals of general medicine: a critical review**. *Biosci Trends* 2009, **3**:202–209.

339. Zwolsman SE, van Dijk N, de Waard MW: **Observations of evidence-based medicine in general practice.** *Perspect Med Educ* 2013, **2**:196–208.

340. Zwolsman S, te Pas E, Hooft L, Wieringa-de Waard M, van Dijk N: **Barriers to GPs’ use of evidence-based medicine: a systematic review.** *Br J Gen Pract* 2012, **62**:e511–21.
